# *Pseudomonas aeruginosa* adapts to octenidine via a combination of efflux and membrane remodelling

**DOI:** 10.1038/s42003-021-02566-4

**Published:** 2021-09-09

**Authors:** Lucy J. Bock, Philip M. Ferguson, Maria Clarke, Vichayanee Pumpitakkul, Matthew E. Wand, Paul-Enguerrand Fady, Leanne Allison, Roland A. Fleck, Matthew J. Shepherd, A. James Mason, J. Mark Sutton

**Affiliations:** 1 Technology Development Group, National Infection Service, PHE Porton, Salisbury, UK; 2 Institute of Pharmaceutical Science, School of Cancer & Pharmaceutical Science, King’s College London, London, UK; 3 Centre for Ultrastructural Imaging, Guy’s Campus, King’s College London, London, UK

**Keywords:** Antimicrobial resistance, Bacterial systems biology

## Abstract

*Pseudomonas aeruginosa* is an opportunistic pathogen capable of stably adapting to the antiseptic octenidine by an unknown mechanism. Here we characterise this adaptation, both in the laboratory and a simulated clinical setting, and identify a novel antiseptic resistance mechanism. In both settings, 2 to 4-fold increase in octenidine tolerance was associated with stable mutations and a specific 12 base pair deletion in a putative Tet-repressor family gene (*smvR*), associated with a constitutive increase in expression of the Major Facilitator Superfamily (MFS) efflux pump SmvA. Adaptation to higher octenidine concentrations led to additional stable mutations, most frequently in phosphatidylserine synthase *pssA* and occasionally in phosphatidylglycerophosphate synthase *pgsA* genes, resulting in octenidine tolerance 16- to 256-fold higher than parental strains. Metabolic changes were consistent with mitigation of oxidative stress and altered plasma membrane composition and order. Mutations in SmvAR and phospholipid synthases enable higher level, synergistic tolerance of octenidine.

## Abstract (web summary)

Bock et al. characterise the adaptation of Pseudomonas aeruginosa to the antiseptic octenidine, using whole genome sequencing, gene expression studies and metabolomics. They attribute this increased tolerance to synergistic changes in efflux and plasma membrane composition via mutations in SmvR, the regulator of MFS efflux pump SmvA, and in phospholipid pathway proteins PssA and PgsA.

## Introduction

Antibiotic-resistant bacteria in healthcare mean increasing reliance on the use of antiseptics and disinfectants (biocides). Studies on high-consequence nosocomial infections caused by *Klebsiella pneumoniae*^1,2^, *Enterococcus faecium*^3^, *Staphylococcus aureus*^4^ and *Pseudomonas aeruginosa*^5^ have shown increased tolerance to biocides, potentially making them problematic for infection-prevention and control (IPC).

*P. aeruginosa*, a Gram-negative opportunistic pathogen, causes outbreaks with high morbidity and mortality in neonatal units^6^, burns patients^7,8^ and persistently in cystic fibrosis sufferers^9^. Multi-drug resistant strains of *P. aeruginosa* are increasing^10,11^, reducing treatment options. Low membrane permeability and multi-drug efflux pumps cause higher biocide tolerance and resistance to many antibiotics^12^.

IPC includes decolonising patients with antiseptic body washes, surface decontamination and hand sanitisation for patients and clinical staff; all rely on biocides effectively killing bacteria. Cationic biocides, such as chlorhexidine, benzalkonium chloride and octenidine, have relatively low toxicity and are effective against many Gram-positive and Gram-negative bacteria^13,14^. Octenidine and chlorhexidine, both gemini-surfactants with two cationic centres linked by an aliphatic hydrocarbon chain, bind to the negatively charged cell membrane, disrupting it and leading to loss of the cell wall^15^. Octenidine then interacts with the inner membrane causing chaotic lipid arrangement and disruption of the cell envelope^16^. Increased biocide tolerance is linked to changes in membrane charge/composition combined with efflux pump overexpression^17^. In *K. pneumoniae*, membrane modification and an upregulated Major Facilitator Superfamily (MFS) efflux pump, combine to mediate resistance to chlorhexidine and cross-resistance to the last-resort antibiotic colistin^2^. Little is known about resistance to octenidine; previous studies have not shown increased tolerance^15,18^.

Previously we showed that *P. aeruginosa* can become stably tolerant to in-use-concentrations of octenidine in laboratory and simulated clinical settings^5^. The adaptation mechanisms were studied here on a genetic and metabolic level, to understand the possible impact of octenidine adaptation in *P. aeruginosa* in the clinic.

## Results

### Stable mutations in an efflux regulator and phospholipid pathways following octenidine adaptation

Stable mutations were identified in octenidine-adapted *P. aeruginosa* strains from various clades (Supplementary Fig. 1) compared to parental strains (Table 1), generated as outlined in Supplementary Fig. 2 and as described previously^5^. All adapted strains (8/8) contained mutations in PA1283, a transcriptional regulator; six out of eight contained SNPs in *pssA* (PA4693), a phosphatidylserine synthase, or *pgsA* (PA2584), a CDP-diacylglycerol-glycerol-3-phosphate 3-phosphatidyltransferase. Additionally, two strains (CAS2 and CAS4) had SNPs in the signal sensor kinase PmrB (PA4777), a two-component regulator involved in biocide and colistin resistance^2,19^. Individual strains (CAS2 and PAO1) had mutations in *hasR* (PA3408), a heme-uptake outer membrane receptor; and PA3328, a FAD-dependent monooxygenase, respectively.

**Table 1:** Mutations present in *P. aeruginosa* strains passaged in increasing concentrations of octenidine followed by 10 passages on TSA in the absence of octenidine.

Parental strain	SmvR (186aa)	PssA (271aa)	PgsA (186aa)	Other	OCT MIC fold increase over parental strain
NCTC 13437	K51R^a^	D240E		SNP in *smvA/R* promoter	256
372261 LCV	R3C	V222G			32
372261 SCV	123^a^			ParS A168V	32
CAS2	Δ43–52	D240G		PmrB S284N, HasR V667I	128
CAS3	A143P	V222G			64
CAS4	Δ82–109		T58M	PmrB T132P	32
GH12	Δ106–109	V222G			64
PAO1	Δ106–109			PA3328 P51H (probable FAD-dependent monooxygenase)	2

*LCV* large-colony variant, *SCV* small-colony variant, *aa* amino acid.

^a^Premature stop codon.

PA1283 is divergently transcribed from PA1282, an MFS transporter, termed *smvA* in *Salmonella enterica* sv. Typhimurium and other organisms^2,20^. PA1283, termed *smvR* for “*smvA*-regulator”, is the TetR-family repressor (TFR) regulating PA1282^2,19^. Mutations in *smvR* included in-frame deletions in four strains (CAS2, CAS4, GH12 and PAO1), premature stop codons in two strains (NCTC 13437 and 372261 small-colony variant (SCV)) and a SNP in the remaining two strains (372261 large-colony variant (LCV) and CAS3). A duplication in *smvR* (base pairs 306–317 and 318–329), coding for amino acids 102–106 (Ala-Ala-Ala-Leu-Met) and 106–110 (Met-Ala-Ala-Leu-Ile), was reduced to a singlet in GH12 and PAO1 deleting amino acids 106–109 (Met-Ala-Ala-Leu). Repeat experiments using the same panel of seven strains, showed this Δ106–109 deletion in *smvR* in 11 of 14 adaptation studies and the deletion of a single alanine (within the triple alanine motif of amino acids 102–104) occurred in 2 of 14 strains (Tables S2-S4).

PssA and PgsA are the first enzymes in divergent phospholipid biosynthetic pathways, starting with the common intermediate CDP-diacylglycerol^21^. Membrane remodelling is a common resistance mechanism, usually linked to lipopolysaccharide (LPS) rather than phospholipids^2,22^. The SNPs in *pssA* encoded V222G (372261 LCV, CAS3 and GH12) and D240E/G (NCTC 13437 and CAS2 respectively). SNPs in *pssA* were found repeatedly in octenidine adaptation experiments with 100% of the population having the alteration V222G in three strains and four further SNPs in this region, including D240V, in a single strain (Supplementary Tables 1-3). Different mutations were found in the same strain background in repeat studies; e.g. PssA-V222G and SmvR-Δ106–109 were found separately in PAO1.

### Efflux pump de-repression precede changes in phospholipid genes

**Fig. 1:**
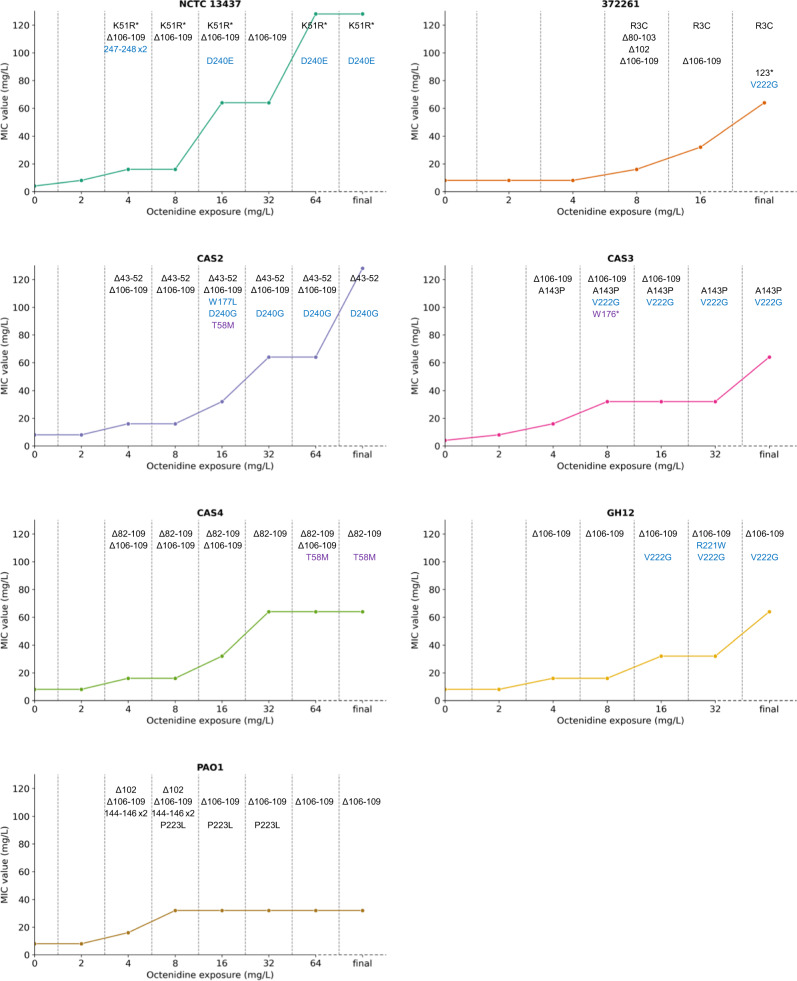
The octenidine tolerance of strain populations increases with exposure to increasing octenidine concentrations and is linked to mutations in SmvR and then PssA/PgsA. Seven strains of *P. aeruginosa* were exposed to doubling concentrations of octenidine as shown on the *x*-axis and in Supplementary Fig. 2. Mutations in SmvR (black) occur at low octenidine concentrations and result in low levels of increased octenidine tolerance. Deletion of amino acids 106–109 occurs in all strains. Mutations in PssA (blue) and PgsA (purple) occur at higher octenidine concentrations and result in higher octenidine tolerance. Mutations were identified using population-mode BreSeq (*P* ≤ 0.01) on PCR products of the genes amplified from populations. ≥10% of the population is listed in the figure, for % of the population containing specific mutations see Supplementary Table 4. *n* = 1 experiment for each strain* premature stop codon; ×2 duplication of listed amino acids; fs frame shift following listed amino acid; final = single colony restreaked 10 times on TSA in the absence of octenidine, for 372261 a large-colony variant (R3C in SmvR and V222G in PssA) and a small-colony variant (123* in SmvR) were picked.

Multiple mutations are commonly seen during antimicrobial adaptation; either sequential changes allowing survival at higher concentrations, or secondary mutations reducing impact on fitness (compensatory)^23^. The presence of multiple mutations in *smvR*, *pssA* and *pgsA* were investigated during adaptation, using population-mode BreSeq^24^ on PCR products, to determine the frequency of each mutation (Fig. 1 and Supplementary Table 4). *smvR* mutations always appeared first, within 2–4 days (0.25× to 0.5× octenidine MIC), corresponding to a two-fold increase in the octenidine population MIC^5^. In-frame deletions within *smvR*, notably deletion of amino acids 106–109, were present in all strains, with the highest percentage (53.8–100%) occurring at passage 2–4 (0.75× to 1× MIC). Strains grown at octenidine concentrations at or above the parental octenidine MIC, acquired SNPs in *pssA* and *pgsA* in six of seven strains (except 372261). The conserved order of the mutations is noteworthy, giving synergistically increased MICs. In some populations, the *smvR* mutations are under-represented in the BreSeq analysis of final populations, notably passage 6 with NCTC 13437 where no *smvR* deletions are observed. This may suggest that these mutations exert a fitness cost when grown in mixed culture environments.

### The same mutations occur in *P. aeruginosa* in a simulated clinical setting

*P. aeruginosa* strains were isolated from a clinically relevant model of mixed-species biofilms in hospital sink traps^5^, exposed four times daily to octenidine handwash (64 days), before the octenidine was removed (27 days) and then reintroduced^5^. Mutations in *smvR*, *pssA* and *pgsA* from *P. aeruginosa* isolates from six timepoints (day 0, 33, 61, 75, 96 and 110) were assessed by population-mode BreSeq analysis (Supplementary Table 5). *smvR* showed multiple mutations in the exposed population, including deletions of amino acids 106–109 and amino acid 102 alone; these mutations again coincided with an increase in the octenidine MIC. When octenidine dosing was paused (day 96), these mutations were not detected, likely because a different sequence type became dominant, but reappeared on reintroduction (day 110). When present, the in-frame deletions appeared at low frequencies within the population (4–8%), except at day 96, where Δ102 was present in 96% of the population. No mutations were observed in *pssA* or *pgsA*; consistent with the previous observation that mutations in these genes only occurred at octenidine concentrations at/above the MIC (maximum octenidine concentration was sub-MIC (~3 µg/mL))^5^.

### Constructing isogenic *smvR*, *pssA* and *pgsA* mutants in *P. aeruginosa*

Isogenic mutations were introduced into PAO1 using a novel recombineering method adapted from *P. putida*^25^ (Supplementary Table 6). Mutations from octenidine-adapted strains (PssA-V222G, PssA-D240G and D240E, PgsA-T58M and SmvR Δ106–109) were introduced into PAO1, and SmvR-Δ106–109 double mutants generated with each PssA/PgsA SNP. Single mutants showed 2–4-fold increase in octenidine tolerance, each SmvR-PssA double mutant showed a 32-fold increase and the SmvR-PgsA double mutant a 16-fold increase, indicating a synergistic effect between mutations in the efflux pump repressor and changes in phospholipid synthesis (Table 2). The increases in MIC for the single SmvR-Δ106–109 mutation was similar to that observed in PAO1, suggesting that the other mutations observed in this background (PA3328 P51H) did not affect susceptibility to octenidine. The MIC increase in the double mutants was lower than those observed for some of the adapted strains, notably NCTC 13437 (256-fold increase). This is likely due to the influence of the strain background, as has been observed in our previous studies^2^. Isogenic strains showed no increase in tolerance to chlorhexidine or other biocides/antibiotics except for single mutants in PssA-D240E and SmvR Δ106–109, which had 2–4-fold increase in MIC for chlorhexidine and alexidine. This differed from the octenidine-adapted strains, where most strains showed elevated resistance to chlorhexidine (6/7 strains), DDAB (2/7) and alexidine (2/7)^5^, likely due to the additional mutations in the adapted strains and/or the influence of individual strain backgrounds. PssA and PgsA variants showed no attributable defect in growth rate or doubling time, compared with wild-type PAO1 and SmvR Δ106–109 alone (although PssA-D240E grew faster) (Supplementary Fig. 3).

**Table 2:** Minimum inhibitory concentrations (mg/L) of a selection of biocides and antibiotics for PAO1 WT and isogenic mutants.

	OCT	CHD	ALEX	DDAB	TOB	AMI	CST
WT	2	4	4	8	8	2	0.5
PssA-V222G	8	4	2	8	8	2	0.5
PssA-D240G	8	4	4	8	8	2	0.25–0.5
PssA-D240E	4	4	4	8	8–16	2	0.5
PgsA-T58M	4	4	2	8	16	2	0.5
SmvR Δ106–109	4	8	4	8	8	2	0.5
PssA-V222G SmvR Δ106–109	64	4	4	8	8	2	0.25–0.5
PssA-D240G SmvR Δ106–109	64	4	4	8	8	2	0.25–0.5
PssA-D240E SmvR Δ106–109	64	16	8–16	8	8	4	0.5
PgsA-T58M SmvR Δ106–109	32	8	4	8	8–16	2	0.5

*OCT* octenidine, *CHD* chlorhexidine digluconate, *ALEX* alexidine, *DDAB* didecyldimethylammonium bromide, *TOB* tobramycin, *AMI* amikacin, *CST* colistin.

All of the single and double mutants were also tested for changes in MIC for existing antibiotics, known to be substrates for one or more RND-family efflux pumps in *P. aeruginosa*. None of the strains showed any significant difference in MIC, compared to the parental strain, with doxycycline, nalidixic acid, meropenem, piperacillin, ceftazidime, ciprofloxacin or chloramphenicol (Supplementary Table 7).

### Octenidine adaptation constitutively increases *smvA* expression

**Fig. 2:**
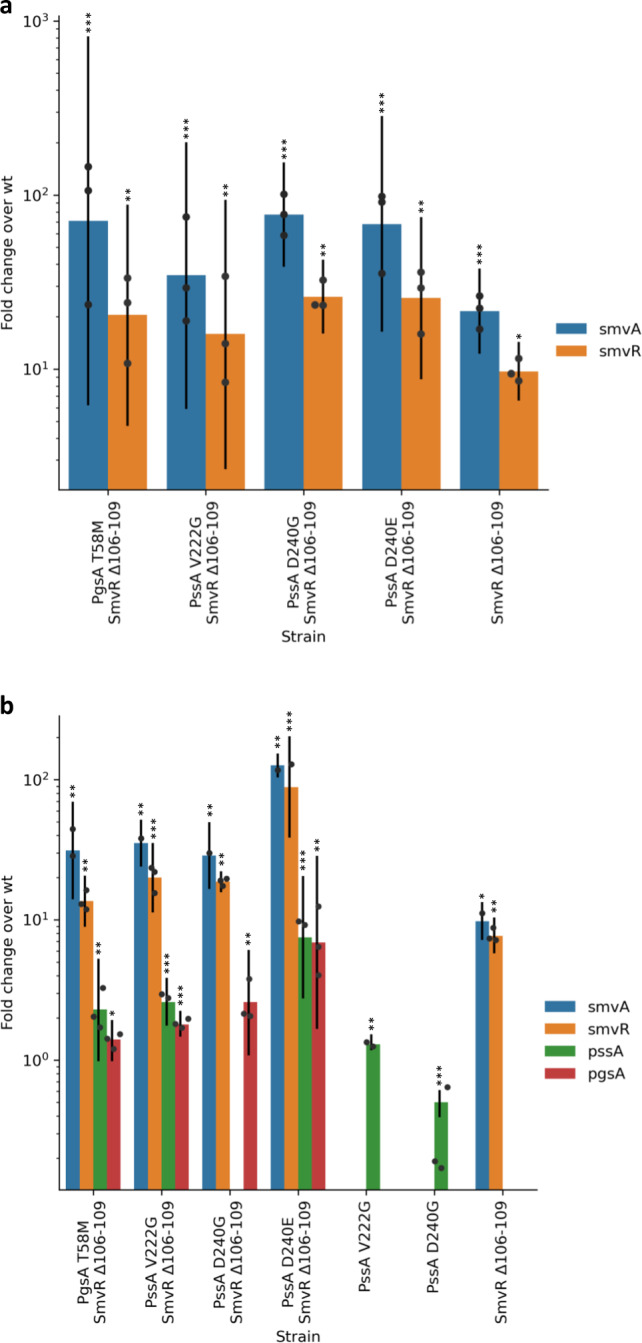
Mutations following octenidine adaptation lead to constitutively expressed *smvA*. Expression levels of *smvA* (blue), *smvR* (yellow)*, pssA* (green) and *pgsA* (red) in isogenic strains of *P. aeruginosa* containing mutations in SmvR (Δ106–109), PssA (V222G, D240G, D240E) and PgsA (T58M), individually or in combination, were compared to wild-type expression levels. No significant changes were detected for *pssA* or *pgsA* (**a**). Expression was also measured following exposure to 0.25× MIC octenidine for 30 min compared to the wild-type strain exposed to octenidine (**b**). Significant RQ values are shown (***P ≤ 0.01, **P ≤ 0.05, *P ≤ 0.1). Error bars represent RQ Min and RQ Max, data points represent means of three technical replicates. Complete results are shown in Tables S9-S10.

Gene expression of *smvA*, *smvR*, *pssA* and *pgsA* was investigated in the isogenic mutants with and without 0.25× MIC octenidine (Fig. 2, Supplementary Tables 8-9). SmvA and SmvR-encoding genes were constitutively upregulated in all strains containing SmvR Δ106–109, in the presence or absence of octenidine, confirming that this mutation de-represses the operon. The expression of *pgsA* was significantly induced by 0.25× MIC of octenidine in all double mutants (1.4–7 fold), while *pssA* expression was induced by octenidine in PAO1 PssA-V222G (1.3 fold) and in most double mutants (2–8 fold), but not in PAO1 PssA-D240G/SmvR-Δ106–109. Levels of *pssA* were repressed in the PAO1 PssA-D240G single mutant (2 fold) compared to the wild type. Relatively small changes in expression of *pssA/pgsA* are consistent with their tight regulation as branch points in the phospholipid biosynthetic pathway^26^, but the mechanism of gene regulation is not known. No significant changes to gene expression of *smvA*, *smvR*, *pssA* or *pgsA* were recorded in the WT PAO1 when challenged with 0.25× MIC octenidine, possibly due to the low challenge dose.

Quantitative PCR of the octenidine-adapted strains also showed that *smvA* and *smvR* were constitutively overexpressed with and without 0.25× MIC octenidine, irrespective of the type of mutation present in *smvR* (Supplementary Table 10). Significantly higher overexpression of *smvA* and *smvR* were observed in all *pssA*/*pgsA* and *smvR* double mutants, compared to the single *smvR* mutant. Interestingly, the PAO1 PssA-D240E/SmvR-Δ106–109 double mutant showed much higher levels of *smvA* and *smvR* expression under octenidine challenge, than the PAO1 PssA-D240G/SmvR-Δ106–109 mutant, suggesting a strong allelic effect of individual SNPs at this position. Changes in *pssA* and *pgsA* expression levels were significant, but overexpression was not observed in all strains, probably due to differing strain backgrounds.

### Single and double *smvR* and *pssA/pgsA* mutations produce distinct changes in central carbon metabolism

A ^1^H high-resolution magic-angle spinning (HR-MAS) NMR approach with multi- and uni-variate analysis, adapted for use with *P. aeruginosa*^27^, was used to detect changes in consumption/excretion of metabolites from isogenic mutants, with and without 0.25× MIC octenidine. This identified: (1) an inducible change in metabolism, occurring in PAO1 WT and all isogenic mutants; (2) constitutive and inducible changes in metabolism associated with some or all of the single isogenic mutants and; (3) a distinct metabolic strategy in the double mutants, not seen in the WT or single isogenic mutants.

**Fig. 3:**
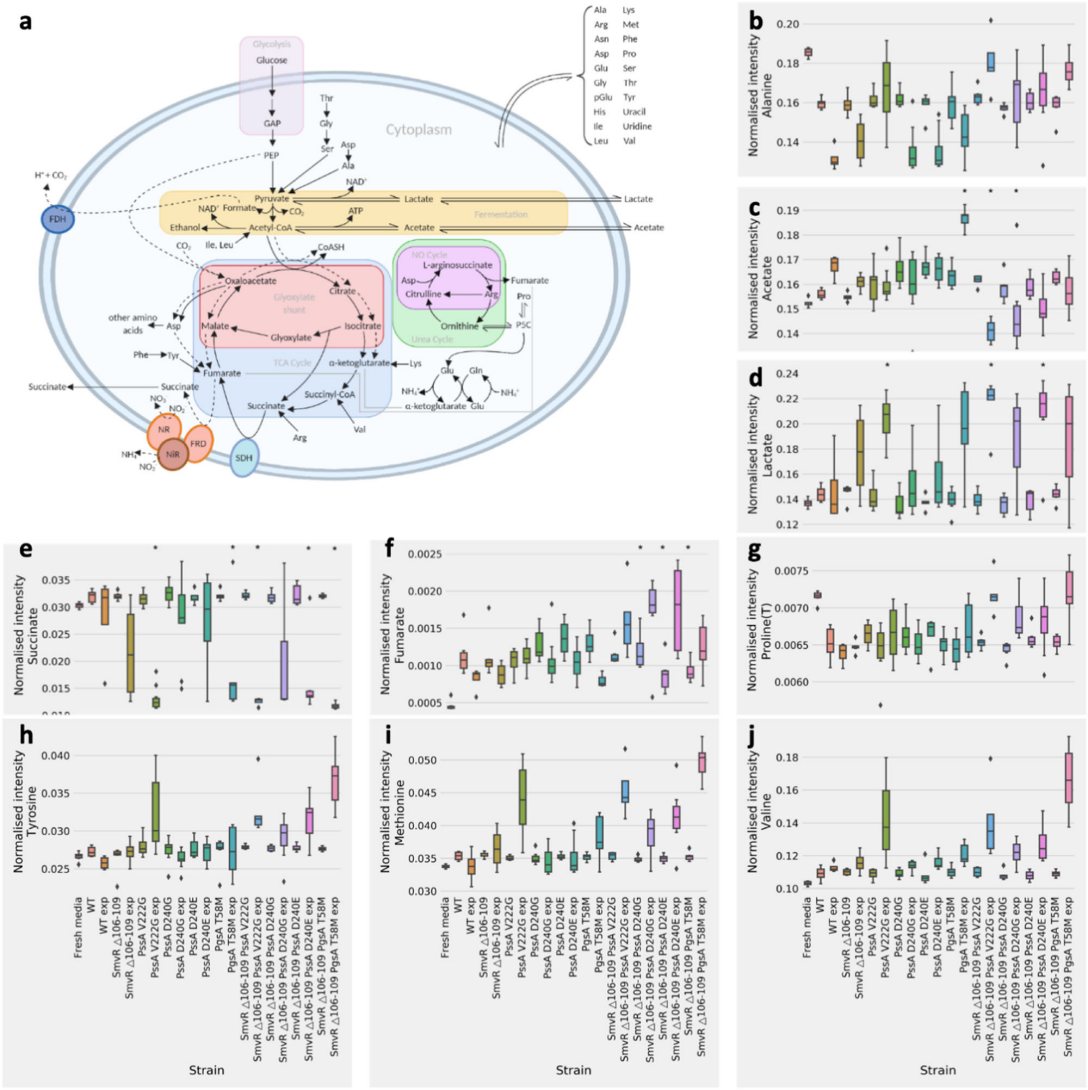
Effect on *P. aeruginosa* PAO1 metabolism of isogenic mutations in the presence/absence of octenidine. The major catabolic pathways of *P. aeruginosa* (**a**). PQN normalised H^1^ NMR resonance intensities, proportional to concentration, of metabolites in fresh or spent media after growth of recombinant PA01 strains to the stationary phase (OD_600_ ~1.2) (**b**–**j**). Exp denotes octenidine exposure within growth medium at 0.25× MIC. For each condition a minimum of *n* = 6 individual colonies were grown in six individual cultures. Asterisk shows significant differences with respect to WT, as determined by one-way ANOVA with Tukey’s post-hoc test (*p* *≤* 0.05). For strains exposed to octenidine (exp), significance is shown with respect to WT exp.

In TSB, *P. aeruginosa* PAO1 relies on arginine and asparagine catabolism and consumption of formate, a key electron donor^28^, during anaerobic respiration. Fermentation pathways are available, but *P. aeruginosa* abstains from utilising glucose, abundant in TSB. For both the WT and all isogenic mutants, octenidine exposure induced a modest increase in formate, glutamate and alanine consumption, triggered glucose, pyruvate, isoleucine and lysine consumption and cytosine, NADH and tryptophan excretion (Fig. 3; Supplementary Figs. 4-5). Limited activation of fermentative pathways was supported by increased production of acetate in WT PAO1 or lactate for certain isogenic mutants.

Single isogenic mutations had little effect on most media metabolites (Fig. 3; Supplementary Figs. 4-5), except the excretion of lactate and consumption of succinate, in all octenidine-exposed isogenic mutants but not WT PAO1. Cellular metabolite analysis reveals that all single mutants of SmvR, PssA or PgsA, showed significant (*p* ≤ 0.05) constitutive increases of acetate and decreases of glutamate levels compared with WT suggesting that acetate is retained in the cell and not excreted. These changes correlated with increases in cellular ethanolamine, ornithine and putrescine; Supplementary Figs. 6-7). Ornithine can be decarboxylated into putrescine and this, together with the downstream product spermidine, can protect the outer membrane from oxidative and antibiotic stress by binding to LPS^29^.

Cellular acetate, glutamate and ethanolamine levels returned to WT levels in all PAO1 SmvR-PssA/PgsA double mutants, with or without octenidine (Fig. 3; Supplementary Figs. 4-5). This implies that the mutations work in synergy, directly or indirectly, to reverse the oxidative-stress effect of each single mutation, and restore the catabolic strategy to WT, or to produce an alternative, constitutive metabolic strategy. The octenidine-inducible consumption and excretion of metabolites to and from the media lends support for the latter. While consumption of succinate and excretion of lactate was similar for double and single isogenic mutants, a distinct metabolic phenotype could be described for the double isogenic mutants on exposure to octenidine, with reduced consumption of alanine and consumption, rather than production, of acetate. There were substantial increases in fumarate, tyrosine, methionine and valine excretion, while proline consumption stopped, consistent with increased succinate dehydrogenase activity and use of the glyoxylate shunt to bypass reactive oxygen species producing steps^30^.

Therefore, both the constitutive and octenidine-inducible metabolic phenotypes of the single and double isogenic *P. aeruginosa* PAO1 mutants are distinct. Oxidative stress is both a constitutive feature of metabolism in the single isogenic mutants as well as being associated with octenidine challenge. In each case, different means are used to overcome oxidative stress.

### Double *smvR* and *pssA/pgsA* mutations induce changes in signatures associated with membrane remodelling

The change in ethanolamine metabolism may be linked to phospholipid biosynthesis. The mutations in the phospholipid pathway enzymes PssA and PgsA, altered cellular lipid metabolism in the isogenic mutants compared to WT PAO1 (Supplementary Figs. 7-10). Changes in acetate and ethanolamine were negatively, and glutamate positively correlated with changes in lipids and N-acetyl-x moieties. In contrast to acetate, glutamate and ethanolamine, which only changed in the single mutants, constitutive changes were detected in resonances assigned to lipid acyl-chain groups; most notably –CH_2_– and –CH_3_ and, to a lesser extent –CH = CH– as well as N-acetyl-x (1, 2 and 3). The identities of the three different N-acetyl groups could not be confirmed using the NMR method. An increase in a second LPS feature (LPS (2)) was identified in the PAO1 SmvR∆106–109/PssA-D240E double mutant only (Supplementary Figs. 7/9). LPS (1) and a resonance assigned to lipid headgroup glycerol (1) suggested an octenidine-inducible effect in the double mutants and WT. This indicates the lipid composition of the plasma and/or outer membrane is altered only when mutations in SmvR are combined with and PssA-V222G, PssA-D240X or PgsA-T58M and may, to some extent, be induced by the presence of octenidine. The change in lipid composition is the most notable change in metabolomic phenotype associated with the double isogenic mutants that display synergistically elevated octenidine tolerance.

Cellular choline and betaine, both of which can act as osmoprotectants^31^, were changed in the isogenic mutants (Supplementary Fig. 5), implying that osmotic stress may play a role in mutations selected by octenidine challenge. Betaine levels were generally lower in the isogenic mutants compared with WT, although PssA-D240X mutants were not affected to the same extent (Supplementary Fig. 5). An increase in choline was observed only in the PgsA mutants (Supplementary Figs. 5/10), implying that the role of choline in the phosphatidylcholine pathway, rather than as an osmoprotectant, had been affected by the PgsA-T58M mutation.

Overall, the octenidine-adapted strains showed broadly similar changes to the same cellular metabolites (Supplementary Figs. 11-13), but in several strains no constitutive (372261 SCV, CAS2, CAS4, PAO1) or inducible changes (CAS4 and GH12) were found. Strains 372261 LCV, CAS3, GH12 and PAO1, showed very few changes whilst in NCTC 13437, 372261 SCV, CAS2, CAS3 there were many changes. This implies that backgrounds and/or other adaptive changes can have a large impact on metabolism. Interestingly, the PAO1 strain with the single SmvR mutation did not show a similar pattern to the isogenic mutant, in terms of either signatures of metabolic stress (acetate, glutamate) or the presence of some of the lipid metabolites (glycerol lipid headgroup (2) and N-Acetyl-x (2)), despite the absence of mutations in PssA/PgsA. It is possible that the additional FAD-dependent monooxygenase mutations in this strain have restored metabolic function.

### Single mutations in SmvR and PssA/PgsA constitutively increase membrane order

**Fig. 4:**
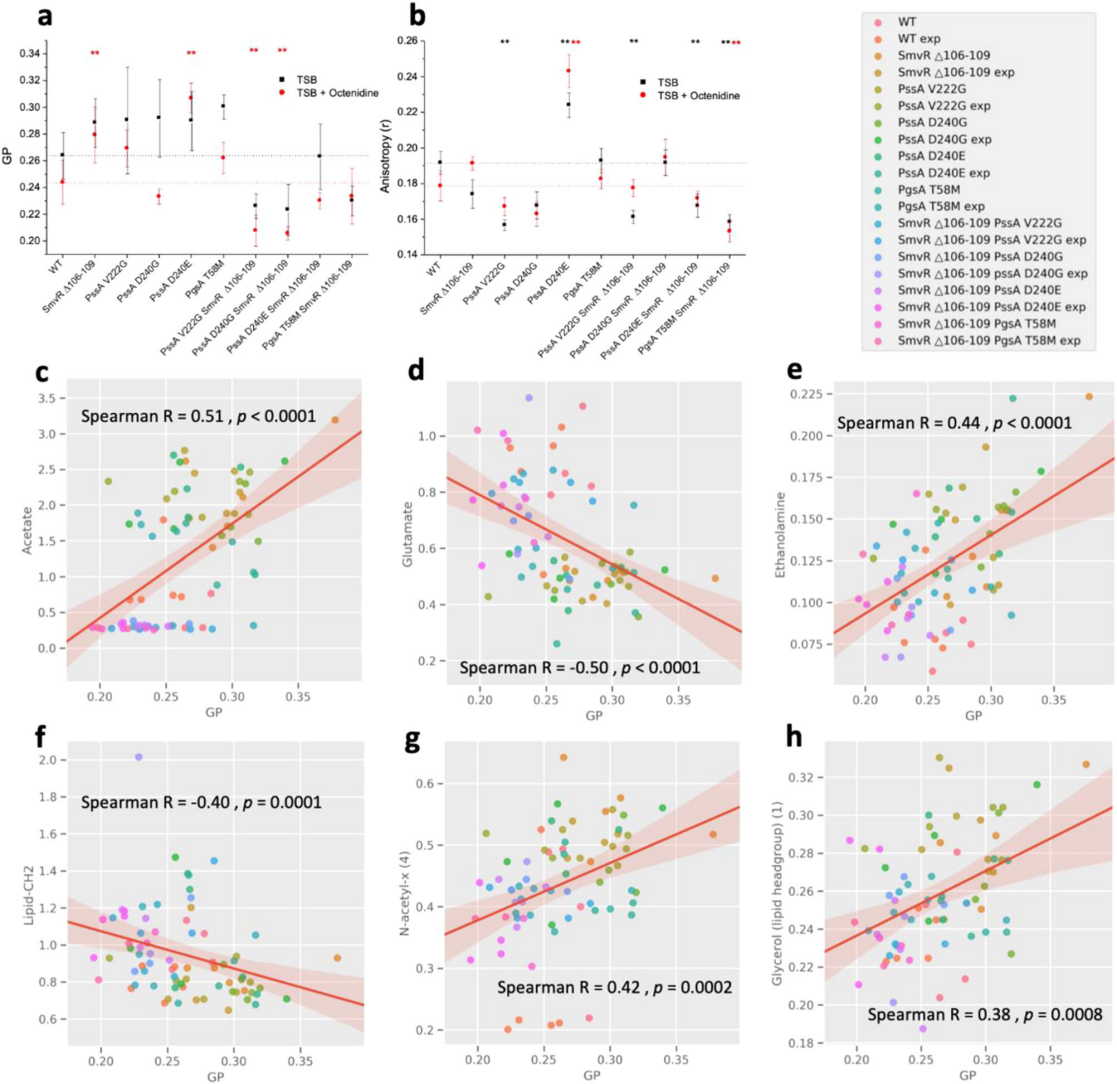
Membrane biophysical parameters in *P. aeruginosa* PAO1 and isogenic strains in the presence/absence of octenidine stress. Measures of lipid order—Laurdan GP (**a**), and lipid fluidity—DPH fluorescence anisotropy (**b**) are shown for fluorescently labelled bacteria grown with or without 0.25× MIC octenidine in TSB. Spearman correlations from a PLS regression model are shown between Laurdan generalised-polarisation (GP) and ^1^H HR-MAS NMR resonance intensity of intracellular metabolites or cell envelope components, which is proportional to their concentration. Recombinant PA01 strains were grown in TSB or TSB with 0.25× MIC octenidine to the stationary phase (OD_600_ ~1.2). Each fluorescence and HR-MAS NMR measurement was carried out on the same biological replicate with a minimum of *n* = 6 biological replicates carried out for each condition. PLS regression models were validated by splitting data into 70/30 training/test sets for use with Monte Carlo cross-validation models. The procedure was run 1000 times to avoid bias by sample separation and model performance was assessed through R2 and Q2 values (Supplementary Tables 13-14). Low GP values correlate with increased lipid –CH_2_ (**f**), lipid –CH_3_ (Supplementary Fig. 16) and glutamate (**d**) and lower levels of acetate (**c**), ethanolamine (**e**) and signals from N-acetyl (4) (**g**) and glycerol in a lipid headgroup (**h**).

Changes in the membrane, suggested by changes in cellular lipid and LPS components, were further investigated using two fluorescent dyes: Laurdan^32^ and diphenylhexatriene (DPH)^33^, both of which are probes reporting on membrane physical properties (Fig. 4). The negligible correlation (R^2^ = 0.00742) between DPH anisotropy and Laurdan Generalized Polarization (GP) indicate that the two molecules report on different effects and, probably different components of the bacterial cell wall (Supplementary Fig. 14). Changes in DPH fluorescence anisotropy were modest although a substantial increase was detected for the PAO1 PssA-D240E single mutant, both in the presence and absence of octenidine (Fig. 4b). Trends could be identified neither for single versus double isogenic mutants nor for individual metabolites and DPH fluorescence anisotropy, using a partial least-squares (PLS) regression model (Supplementary Fig. 15).

GP was sensitive to both constitutive and induced changes in membrane order (Fig. 4a). All single SmvR and PssA/PgsA mutants showed a similar, modest increase in GP, indicating a more ordered membrane^32^ compared with WT PAO1. Double mutants, however, showed decreased GP compared to PAO1 indicating a more disordered membrane. The addition of 0.25× MIC octenidine decreased order in all strains except PssA-D240E, in which it increased slightly. PLS regression of GP and relative cellular metabolite levels in the isogenic strains (R^2^ = 0.429, Q^2^ = 0.218; Supplementary Fig. 16) identified seven metabolites, whose concentration correlated with the change in Laurdan GP (*p* < 0.05) (Fig. 4c-h; Supplementary Fig. 16). Metabolites likely associated with oxidative stress (acetate, glutamate, ethanolamine and putrescine) correlated most strongly, followed by changes in lipids and an unidentified N-acetyl resonance (lipid CH_2_, lipid CH_3_, N-acetyl-x (1 and 4), glycerol (lipid headgroup) (1), LPS (2 and 3)) (Fig. 4; Supplementary Fig. 16). Changes in lipid order are shown to affect or be affected by both the metabolism of the cell and, more specifically, by lipid metabolism. The relationship between these changes and increased octenidine tolerance is discussed below.

Substantially decreased membrane order was observed for octenidine-adapted NCTC 13437, 372261 LCV and CAS2, whereas the parental strains did not show any induced effect on membrane order (Supplementary Fig. 17).

Transmission electron microscopy did not show gross differences in morphology between single and double mutants, although there was a trend towards a greater frequency of elongated (Supplementary Fig. 18) and septating (Supplementary Fig. 19) cells in the PAO1 PssA-D240G/SmvRΔ106–109 double mutants compared with the corresponding single mutants and wild-type strain, but only in the presence of octenidine.

## Discussion

**Fig. 5:**
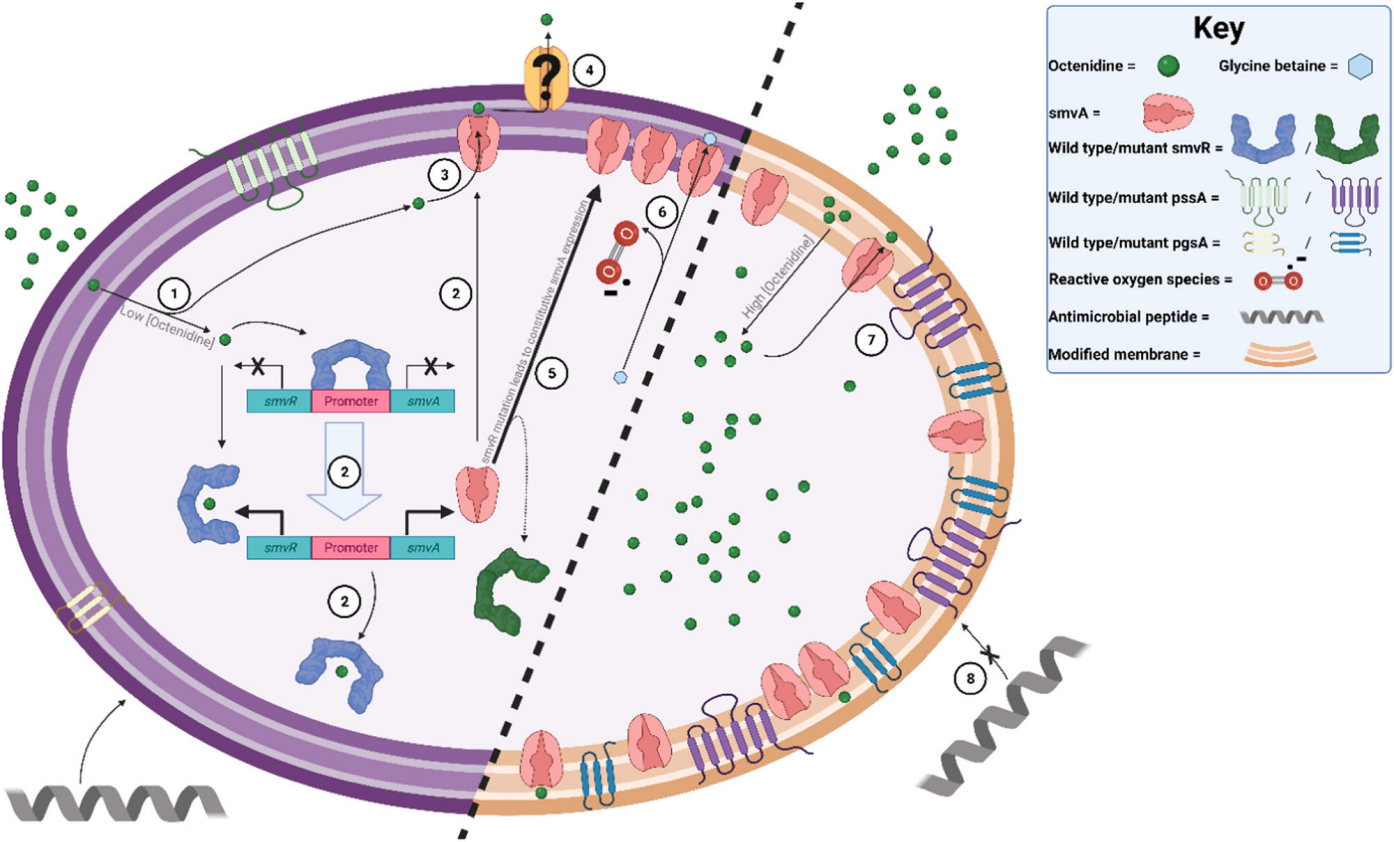
Schematic summary of results and hypotheses. (1) A low concentration (<MIC) of octenidine enters the cell in which SmvR is bound to the promoter of *smvAR*; (2) Octenidine binds to SmvR releasing *smvAR* promoter, *smvR* and *smvA* are increasingly transcribed, SmvR is bound by octenidine, SmvA is inserted into the inner membrane; (3) SmvA effluxes octenidine into the periplasmic space; (4) Octenidine is effluxed through the outer membrane by an unknown mechanism, possibly by redundant pumps (e.g. OprJ, OprM, OprN, OmpD); (5) Mutations in *smvR* lead to constitutive expression of SmvA, (6) Overexpressed SmvA also effluxes glycine-betaine cations leading to oxidative stress, which destabilises the membrane; (7) At increased octenidine concentrations (≥MIC) mutated PssA and PgsA modify the membrane, changing fluidity, order and charge with the following possible consequences: more stable SmvA or support with folding; more stable membrane under octenidine stress; less cation/octenidine binding or increased cation/octenidine release; increased proton gradient supporting glyoxylate shunt, increased efflux by SmvA and decreased oxidative stress; (8) Increased resistance to cationic antimicrobials may lead to increased resistance to antimicrobial peptides.

Mounting evidence of decreased susceptibility to biocides commonly used in healthcare is concerning given increased reliance on them to prevent infections with MDR organisms. In this study we find that stable, increased tolerance to octenidine in *P. aeruginosa* is caused by increased efflux and membrane modifications, with higher levels achieved where these two mechanisms of resistance work in synergy (Fig. 5).

Increased expression of efflux pumps is a common resistance mechanism for antibiotics and biocides^17^. Stable mutations in regulators of multiple efflux pumps, commonly include TFRs, regulating MFS pumps (equivalent to SmvR) (e.g. QacR in *Staphylococcus aureus*)^34,35^. We have previously shown that the homologue of SmvR, the regulator of the MFS efflux pump SmvA, is mutated in response to chlorhexidine exposure in *Klebsiella pneumoniae*, *Klebsiella oxytoca*, *Citrobacter freundii* and *Salmonella enteritidis*, leading to de-repression of SmvA^2,19^. SmvA itself is mutated in octenidine-adapted *K. pneumoniae*^19^. We found that the regulator SmvR was always mutated and no mutations were observed in SmvA in octenidine-adapted *P. aeruginosa*; this likely reflects different substrate binding between efflux pumps in different species. Deletion of a 12 bp nucleotide tandem repeat (GGCGGCGCTGAT) encoding amino acids 106–109 (Met-Ala-Ala-Leu), outside of the DNA binding region in the canonical TFR, was found in all strains tested; this resulted in at least 20-fold upregulation of SmvA and SmvR (Fig. 5). Tandem repeats are disproportionately frequent in genes whose products are involved in stress response, allowing for an immediate response by mechanisms including slip strand mispairing or polymerase slipping^36^. Indeed, we found that the removal of one of the 12 bp tandem repeats in SmvR always occurred as an initial mutation, within 2–4 days following exposure to low levels of octenidine, in all strains tested. As this was seen in both lab-adaptation studies and repeatedly in selected isolates from the sink trap model^37,5^, this is likely to be a common initial response of *P. aeruginosa* to low levels of octenidine and/or other stresses (Fig. 5). The multiple mutations found in SmvR, all of which affect the regulation of SmvA, also suggest this is a hypermutable region, allowing the organism to adapt quickly to stress.

In *K. pneumoniae*, docking studies showed that SmvA interacts with both chlorhexidine and octenidine as potential substrates^19^. *P. aeruginosa* SmvA shows 40% homology with *K. pneumoniae* SmvA which, taken together with its upregulation following octenidine adaptation, suggests SmvA may transport cationic biocides in *P. aeruginosa*, also (Fig. 5). However, SmvA was not significantly upregulated in WT PAO1 in the presence of 0.25× MIC octenidine, differing from *K. pneumoniae* where clear upregulation was observed^2,19^. Higher levels of octenidine may, however, show an increase in *smvA* transcription. PAO1 SmvR transposon mutants showed an increase in chlorhexidine MIC, but not octenidine (Supplementary Table 11), suggesting possible differences in substrate specificity of the two pumps/regulators. Although there is clear evidence for SmvA’s role in mediating cationic biocide resistance in different Gram-negative bacteria, questions remain around how this affects efflux of compounds out of the cell. In *Salmonella*, SmvA is proposed to work in conjunction with one or more outer membrane proteins, such as TolC and/or OmpW, to mediate export of the substrate methyl viologen^38^. We did not see significant changes in MIC or MBC for octenidine or chlorhexidine, using transposon mutants in any of the characterised outer membrane proteins associated with efflux pumps in PAO1 (OprJ, OprM, OprN, OpmD; Supplementary Table 11). This suggests that if there is an outer membrane component involved in SmvA-mediated efflux, it is not within this set of known OMP efflux components or that the function is redundant, with no single OMP mutant having a noteworthy impact on MIC (Fig. 5). Other efflux pumps have been previously implicated in mediating resistance to chlorhexidine, notably MexCD-OprJ^39^. However, a PAO1 derivative overexpressing MexCD-OprJ due to an insertion in *nfxB* (strain K1536^39^) did not show an increased MIC for octenidine (Supplementary Table 11).

Betaine levels were decreased in most single and double mutants, with a significantly increased expression of SmvA, in the presence and absence of octenidine. Choline-derived, glycine-betaine can be accumulated in cells as an osmoprotectant^31^. SmvA was originally characterised as a pump for methyl viologen^20^, a small di-cation, and subsequent studies demonstrated a role in mediating increased resistance to other cationic antiseptics and biocides^2,19^. It is possible that glycine-betaine, itself a quaternary ammonium cation, may also be effluxed by SmvA, resulting in reduced levels of betaine in SmvA-overexpressing strains (Fig. 5). Choline levels were only modified in isogenic mutants with mutations in PgsA, most likely as a consequence of altered phosphatidylcholine metabolism. Further studies of SmvA substrate specificity will confirm whether SmvA effluxes a range of di-cations including octenidine, chlorohexidine and/or glycine-betaine in *P. aeruginosa*.

Mutations in phospholipid biosynthetic genes also affect octenidine tolerance of *P. aeruginosa*. The function and regulation of PssA and PgsA have been well described in *E.coli*^21,26^. PssA (phosphatidylserine synthase A) converts the common intermediate, CDP-diacylglycerol, to phosphatidylethanolamine (PE), whereas PgsA (phosphatidylglycerophosphate synthase A) is the first step in the parallel pathway generating phosphatidylglycerol (PG). *P. aeruginosa* PgsA shows high protein homology (60%) with *E. coli* PgsA, whereas PssA shows low conservation outside of the active PssA domain (overall 29% protein sequence homology) with six predicted transmembrane domains present only in *P. aeruginosa* PssA. This suggest a different mechanism of regulation to that seen for *E.coli* PssA, which transiently associates and dissociates with acidic phospholipids in the membrane^40^. Residues consistently mutated in *P. aeruginosa* PssA, V222 and D240, are outside of the CDP-alcohol phosphatidyltransferase domain and located either side of the fifth predicted transmembrane domain. As PssA regulation appears different in *P. aeruginosa*, we cannot define whether the mutations directly affect enzyme function by modulating interactions with the membrane, or influence expression/transcript stability of this pivotal enzyme. The T58M SNP in PgsA is within the predicted CDP-alcohol phosphatidyltransferase domain and may directly influence enzyme activity.

Cell surface modification is a common mechanism of resistance to cationic compounds, linked especially to modified lipid A and a more neutral membrane that may decrease binding of the cations^22,41^. Changes to the phospholipid composition have not been previously described as a mechanism of cationic biocide/antimicrobial peptide (AMP) resistance in Gram-negative bacteria. Studies in *S. aureus* have shown phospholipid composition modulates AMP resistance by increase or decrease in membrane fluidity or order^42^. In *S. aureus*, resistance to daptomycin, a membrane-active lipopeptide antibiotic analogous in function to cationic biocides, is caused by a loss of function mutation in PgsA^43^ or gain of function mutations in cardiolipin synthase^44^, resulting in a net decrease in anionic PG in the membrane, due to accumulation of glycolipids and phosphatidic acids upstream of PgsA. The mutations in *P. aeruginosa* PgsA and PssA are also likely to affect the ratio of phospholipids in the membrane, possibly also leading to a decrease of PG (Fig. 5). What the exact membrane changes are and whether these changes directly affect binding/release of octenidine from the bacteria, consistent with recently described mechanistic descriptions of octenidine activity^16^ or mediate effects indirectly by modulating function of one or more membrane proteins (including SmvA) remains to be determined.

Surprisingly, mutations in PssA and PgsA have a similar metabolomic effect on the cell to those of SmvR mutations. Individually, they give only 2–4-fold increase in octenidine tolerance but these were synergistic when combined, leading to elevated resistance. All isogenic single mutants showed a more ordered membrane compared to WT, although this may be achieved through different mechanisms. Constitutive changes in carbon metabolism present in the single mutants were lost in the double mutants, and membrane order decreased, especially in the presence of octenidine. Although this is reminiscent of the WT, the double mutants respond very differently to octenidine challenge compared with either the WT or single mutants, consistent with an enhanced glyoxylate shunt. In octenidine-adapted strains, mutations in SmvR always occurred first and mutations in PssA or PgsA were never observed in isolation; these mutations only occurred at octenidine concentrations higher than the WT MIC, in the presence of overexpressed SmvA (Fig. 5). Changes in acetate and glutamate levels (consistent with dissipation of constitutive, oxidative stress) and a decrease in membrane order to wild-type levels, supports the hypothesis that these mutations are compensatory, rather than simply being driven by increased selective pressure. Alone, increased numbers of SmvA pumps may not have the proton gradient to efflux effectively, functioning as a proton antiporter, while PssA and PgsA alteration of lipid environment may confer only limited resistance to octenidine. Together, PssA/PgsA may create an improved membrane environment for glyoxylate shunt activity, increasing proton gradients and enhancing the efflux activity of SmvA. The inducible nature of the changes in metabolism associated with this pathway support the hypothesis of enhanced efflux, as without octenidine presence, maintenance of the proton motive force would not be required.

Apart from an elevated glyoxylate shunt in response to octenidine challenge, the change in lipid composition is the most notable metabolomic phenotype associated with the double isogenic mutants. This indicates that the lipid composition of the plasma membrane is only stably altered when mutations in SmvR and one of PssA-V222G, PssA-D240X or PgsA-T58M are combined. Changes in lipid acyl resonances, with increases of –CH = CH–, –CH_2_ and –CH_3_ in the double mutants, correlated with the decreases in membrane order compared to the single mutants. Changes in phospholipid composition and membrane order associated with altered headgroup or saturation and length of acyl chains, are known to affect membrane protein folding and consequently protein function^45,46^ including both efflux pumps and succinate dehydrogenase^47^. MFS efflux pump LmrP in *E.coli* was found to depend on the presence of PE to form structural intermediates required for the transport process^48^. The dimerisation of EmrE, an *E. coli* SMR efflux pump, into active transporters is also affected by the lipid composition of the membrane^45^. Given the evidence of direct interactions between membrane proteins and specific phospholipids and assuming the large increases in *smvA* transcript translates into concomitant increases in protein content, it is likely that overexpression of the efflux pump also influences membrane lipid structure and vice versa (Fig. 5).

We have shown that depressed efflux pump expression in synergy with lipid modification, increase the tolerance of *P. aeruginosa* clinical isolates to octenidine. This is the first time that this mechanism of synergy has been demonstrated and the first time that stable adaptation to octenidine has been described. We do not see loss of fitness of double mutants or adapted strains as shown by growth curves and *Galleria mellonella* infection^5^. We found the same TFR mutations in our simulated clinical setting, but we did not find mutations in phospholipid synthesis pathways, under the conditions used, likely due to lower (sub-MIC) selective pressure in the sink trap model^5^. In other clinical settings, such as during decolonisation of the skin using octenidine at 0.3% (3 mg/mL), *P. aeruginosa* might be exposed to higher octenidine concentrations, possibly leading to selection pressure for mutations in both *smvR* and phospholipid biosynthetic genes. This could lead to the type of synergistic adaptations we have described here and elevated levels of octenidine tolerance. We did not find any indication of cross-resistance to antibiotics in our isogenic mutants, contrasting with previous results showing that octenidine adaptation can select mutants with increased resistance to clinically relevant antibiotics through mutations in other membrane modification systems, such as PmrB (as seen in octenidine-adapted Cas2 and Cas4)^5^. Acyl-chain remodelling e.g. saturation and chain length as induced here, is also a known mechanism of resistance to antimicrobial peptides AMPs^49^, and we cannot rule out the possibility that cross-resistance to such host-defences might also result from this type of selection (Fig. 5).

## Methods

### Bacterial strains and reagents

The *P. aeruginosa* strains are shown in Supplementary Table 12 and a phylogenetic tree in Supplementary Fig. 1. Transposon mutants came from the Manoil lab^50,51^ and from Keith Poole (Queen’s University, Canada). The adaptation process was as described in Shepherd et al.^5^ and Supplementary Fig. 2 and was carried out twice. In brief, a selection of 7 reference (PAO1) and MDR strains, representative of the two main groups of *P. aeruginosa* strains defined previously^52^ were selected for the study. Strains were exposed to octenidine dihydrochloride in Tryptic Soy Broth (TSB), starting at 2 µg/mL (0.25–0.5× MIC) and grown for 2 days prior to subculture in double the concentration of octenidine, until the strain either failed to grow or the concentration reached 64 µg/mL. Populations from these studies were sampled throughout the passaging and as a final population and these were used for BreSeq analysis (see below). Strains were serially passaged 10 times on Tryptic Soy Agar (TSA) plates in the absence of octenidine. The minimum inhibitory concentration of octenidine for single colonies was checked and isolates showing elevated resistance to octenidine (for 372261 a small-colony variant, SCV, and a large-colony variant, LCV) were sent for whole-genome sequencing.

All strains were grown at 37 °C on TSA or TSB (Oxoid, 1896417, UK), unless specified. Biocides and antibiotics used in this study [octenidine dihydrochloride, chlorhexidine digluconate, didecyldimethylammonium bromide (DDAB), alexidine dihydrochloride, amikacin, colistin and tobramycin] were purchased from Sigma–Aldrich (Steinheim, Germany), except octenidine dihydrochloride (supplied by Schülke & Mayr, Norderstedt, Germany).

Exposure of sink trap populations to octenidine, isolation and characterisation of *P. aeruginosa* strains was described previously^5^. In brief, a sink trap taken from a UK hospital was flushed for 30 s four times a day at a flow rate of 4 L/min with the addition of 2 mL octenidine bodywash containing 0.3% octenidine. The octenidine dosing continued for 63 days, was paused for 27 days and then resumed for a further 20 days. 100 µL of water from the sink trap were plated on cetrimide-nalidixic acid agar (Oxoid Ltd, Basingstoke, UK) and incubated at 37 °C overnight to select for *P. aeruginosa*. Cell populations from days 0, 33, 61, 75, 96 and 110 were used for MIC and BreSeq analyses.

### Minimum inhibitory concentration

The minimum inhibitory concentrations (MICs) were measured using the broth microdilution method in polypropylene plates (Greiner) as described previously^1^. One hundred microlitres of 1 × 10^5^ colony-forming units/mL in TSB were mixed with 100 µL of the diluted biocide/antibiotic, and optical density at a wavelength of 600 nm (OD_600_) was measured after 24 h of static incubation at 37°C. MIC was defined as the lowest concentration of biocide/antibiotic at which no growth was observed (OD_600_ < 0.1 after subtracting the blank).

### Next-generation sequencing and sequence analysis

DNA was purified using a Wizard genomic DNA purification kit (Promega, Wisconsin, US). DNA was tagged and multiplexed with the Nextera XT DNA kit (Illumina, San Diego, US) and sequenced by PHE-GSDU (Public Health England Genomic Services and Development Unit) on an Illumina (HiSeq 2500) with paired-end read lengths of 150 bp. A minimum 150 Mb of Q30 quality data were obtained for each isolate. FastQ files were quality trimmed using Trimmomatic^53^. SPAdes 3.1.1 was used to produce draft chromosomal assemblies, and contigs of less than 1 kb were filtered out^54^. FastQ reads from octenidine-exposed isolates were subsequently mapped to their respective WT pre-exposure chromosomal sequence using BWA 0.7.5^55^. Bam format files were generated using Samtools^56^, and VCF files were constructed using GATK2 Unified Genotyper (version 0.0.7)^57^. They were further filtered using the following filtering criteria to identify high-confidence SNPs: mapping quality >30; genotype quality >40; variant ratio >0.9; read depth >10. All the above-described sequencing analyses were performed using PHE Galaxy^58^. BAM files were visualised in Integrative Genomics Viewer (IGV) version 2.3.55^59^. Whole-genome alignment and phylogenetic tree generation were performed using progressive alignment in Mauve Version 20150226 build 10. Tree visualisation was performed in FigTree Version 1.4.3.

### BreSeq

Colony PCR using primers listed in Supplementary Table 6 (LBOL191 and 192 (*pssA*), LBOL193 and 194 (*smvR*), and LBOL315 and 316 (*pgsA*)) to amplify the complete genes was performed on total cultures for each passage and strain. PCR products were purified (Qiagen PCR purification kit, Hilden, Germany), eluted in 30 µL ddH_2_O and quantified using Invitrogen™ Qubit™ Fluorometer dsDNA kit. To increase read depth coverage, PCR products from all three reactions per time point and strain were mixed to contain 20 µg/mL DNA from each reaction and sent for Illumina Sequencing at Public Health England NGS sequencing service. Resulting contigs with a coverage of >15k were compared to the gene sequence of PAO1 using BreSeq polymorphism mode^60^ with a cut-off *P*-value of 0.01 to identify the percentage of the population that had a given SNP or deletion over time. This method uses a maximum likelihood analysis to identify the prevalence of SNPs or small deletions within a bacterial population. Each result was manually checked for false positives according to the BreSeq protocol on the Barrick lab website (http://barricklab.org/twiki/pub/Lab/ToolsBacterialGenomeResequencing/documentation accessed between October 2017 and June 2019).

### Recombineering

The construction of isogenic strains in *P. aeruginosa* was adapted from Aparicio et al.^25^ and Nyerges et al.^61^. PAO1 was selected for the recombineering work due to its relative drug sensitivity and availability of resistance markers for maintenance of plasmids. Oligos (Supplementary Table 6) were designed using MODEST^62^ to include single SNPs or a short 12 bp deletion. Standard desalted ultramers were from Integrated DNA Technologies (IDT, Leuven, Belgium) with phosphorotioate bonds on the first two and last two nucleotides. PAO1 cells were made electrocompetent by washing three times in 300 mM sucrose before being transformed with pSEVA658-ssr^63^ (Ec2 setting: 2 mm-gap cuvette, 2.5 kV, 25 µF, 200 Ω, in a BioRad MicroPulser, Watford, UK) and selected on 30 µg/mL gentamicin/TSA. A fresh colony was inoculated into TSB/gentamicin. The overnight culture was diluted to OD_600_ 0.2 in 20 mL TSB/gentamicin. At OD_600_ 0.4–0.5 1 mM XylS effector 3-methylbenzoate (3MB) was added for 30 min at 250 rpm and 37 °C to induce expression of Ssr. Cells were made electrocompetent and 1 µL 100 µM oligo transformed as above. Colonies were selected on 512 µg/mL octenidine TSA plates. Resulting colonies were screened by Sanger Sequencing (SNPs, Genewiz, UK) or meltcurve analysis (12 bp deletions) using primers listed in Supplementary Table 6. Meltcurve analysis (60.0–95.0 °C recorded every 0.3 °C) was performed on a StepOne Plus (Applied Biosystems, UK). Positive colonies were checked for off-target mutations via NGS (see above). Double mutants were constructed by recombineering LBOL365 into already constructed *pssA*/*pgsA* SNP mutants still containing pSEVA658-ssr. Mutants were regularly checked for reversion by meltcurve and Sanger Sequencing.

### Growth curves

Three single colonies grown in TSB at 37 °C 250 rpm overnight were diluted to OD_600_ 0.01 in TSB and observed at OD_600_ every 30 min for 24 h at 37 °C static growth. Differences in doubling time and intrinsic growth rate were calculated using growthcurver in R version 3.5.1, ANOVA was performed in in GraphPad Prism version 6.04 for Windows, GraphPad Software, La Jolla California USA.

### RNA extraction and quantitative PCR

qPCR was used to measure the expression of *pssA*, *pgsA*, *smvR* and *smvA* in the octenidine-adapted and isogenic PAO1 mutants using primers listed in Supplementary Table 6 as described previously^1^. Triplicate overnight cultures grown in TSB were back diluted to an OD_600_ of 0.1, and exposed to 0.25× MIC octenidine or no octenidine for 30 min. Cells were then harvested using RNA protect bacteria reagent (Qiagen, Hilden, Germany) at mid-log phase (OD_600_ 0.5), and RNA extracted using the RNeasy minikit (Qiagen), including on-column DNase treatment according to the manufacturer’s instructions. In addition, 5 µg RNA was treated with a DNA-free kit (Ambion, UK), of which 0.2 µg RNA was reverse transcribed using the SuperScript III first-strand synthesis system for RT-PCR (Invitrogen, UK) according to the manufacturer’s instructions. Samples were checked for DNA contamination by performing qPCR on a negative reverse transcription reaction for each sample. qPCR was carried out in at least triplicate on each sample using a StepOnePlus real-time PCR system (Applied Biosystems, UK) and Fast SYBR green master mix (Life Technologies, UK). Data were analysed using Expression Suite Software version 1.1 (Life Technologies, UK) using *fabD* and *rpoD* as endogenous controls and taking primer efficiency into account. Changes in expression are in comparison to the isogenic WT strain, where those exposed to 0.25× MIC octenidine are compared to WT exposed to 0.25× MIC octenidine, and those not exposed are compared to WT not exposed. Comparison of WT exposed and unexposed showed no significant changes in gene expression.

### HR-MAS including for supernatant

Individual colonies of *Pseudomonas aeruginosa* strains selected from TSA plates were grown in 10 ml TSB, containing sub-inhibitory octenidine (¼ MIC) when applicable, in 50 mL centrifuge tubes at 37 °C overnight (~16 h) without shaking. Optical density was measured by absorbance at 600 nm to ensure consistent growth to the stationary phase (OD ~1.2). The bacterial suspension was pelleted by centrifugation at 4000 × *g*, 4 °C for 10 min. The supernatant was sterilised by addition of 0.01% (w/v) sodium azide and D_2_O containing 2,2,3,3-D_4_-3-(Trimethylsilyl) propionic acid sodium salt (TMSP-2,2,3,3-D_4_) was added to a concentration of 10% (v/v) to provide a deuterium lock and reference signal. The pellet was washed twice with PBS and snap frozen using liquid nitrogen and dehydrated by freeze drying. Liquid state ^1^H NMR spectra of spent media samples were acquired at 298 K and 600 MHz for ^1^H on a Bruker Advance II 600 NMR spectrometer (Bruker Biospin, UK) with a 5 mm QCl helium-cooled cryoprobe and a refrigerated SampleJet sample changer to keep samples at 4 °C prior to acquisition. 1D CPMG-presat (cpmgpr1d) experiments were acquired with 32 scans and a spectral width of 19.8 ppm at a temperature of 298 K. 2D ^1^H correlation spectroscopy (COSY) and ^1^H–^13^C Heteronuclear Single Quantum Correlation (HSQC) experiments were performed on representative datasets to assist metabolite assignment using Bruker settings. For HR-MAS NMR Lyophilised pellets cell pellets were rehydrated with 40 μl D_2_O containing TMSP. Rehydrated pellets were placed inside Kel-F inserts (Bruker, B4493), which were inserted into 4 mm Magic Angle Spinning (MAS) rotors (Bruker, P/N H14355). All 1D spectra were recoded using the Bruker cpmgr1d spin echo pulse with water presaturation delay of 1 second with a spectral width of 16.02 ppm and a ^1^H 90 pulse length was around 12 ms. HR-MAS NMR acquisition was carried out at 310 K with an initial 8 dummy scans and 64 acquisition scans. The free induction decay was multiplied by an exponential function with 0.293 Hz line broadening. Spectra were phased manually, and their bassline corrected automatically using Topspin (Bruker). HR-MAS NMR 2D homonuclear shift correlation with presaturation during relaxation delay. ^1^H COSY (cosypqrf) and ^1^H–^13^C correlation HSQC (hsqcpc) spectra were also collected using standard Bruker pulse sequences to aid metabolite assignment. All spectra were referenced to 0 ppm using the TSP reference peak. Metabolites were assigned to NMR peaks using a combination of literature, the biological magnetic resonance data bank (BMRB) metabolite database and Chenomx profiler software (Chenomx, Edmonton, Canada). Assignments were confirmed using 2D ^1^H COSY and natural abundance ^13^C HSQC experiments. All spectra were normalised using probabilistic quotient normalisation prior to analysis. Volcano and box and whisker plots were created using the python modules numpy, pandas, scipy and seaborn. Volcano plots provide a global view of all significant, relative changes in metabolites in a given condition while box and whisker plots reveal the change in a particular metabolite across the full range of isogenic mutants in octenidine unchallenged and challenged conditions. Fold change was calculated by taking the ratio of peak intensities from the two sets of data being compared (treatment/control). *P* values were calculated using the Mann–Whitney U test to compare means of the treatment and control and a false rate was set using the Benjamini–Hochberg method (α = 0.05). Partial least square regression was performed using the scikit learn python module using one component. Data were split into 70/30 training/test sets and used for Monte Carlo cross-validation models. The procedure was run 1000 times to avoid bias by sample separation and model performance was assessed through R^2^ and Q^2^ values (Supplementary Tables 13-14). Correlation between metabolite intensities and fluorescence measurements for the same biological replicate were assessed using the Spearman coefficient.

### Laurdan/DPH

*Pseudomonas aeruginosa* strains were cultured as described for HR-MAS/spent media NMR methods and bacterial suspension was fixed by addition of formaldehyde to a concentration of 0.25%. After fixation, cells were washed three times with PBS before resuspension in PBS for data acquisition. Laurdan fluorescent dye or 1,6-diphenyl-hexa-1,3,5-triene (DPH) was added to a final concentration of 2.5 μM and samples were incubated with either dye for one hour prior to acquisition with minimal exposure to light. For acquisition, samples were placed in a quartz cuvette and measurements taken using a Varian Cary Eclipse fluorescence spectrometer (Mulgrave, Victoria, Australia). Laurdan fluorescence measurements were taken by excitation at 350 nm and emission was detected between 400 and 600 nm. General polarisation was calculated using the following equation where I_440_ and I_490_ stands for intensity at 440 and 490 nm, respectively:

$${{{GP}}}=\frac{{{{{{{\rm{I}}}}}}}_{440}-{{{{{{\rm{I}}}}}}}_{490}}{{{{{{{\rm{I}}}}}}}_{440}+{{{{{{\rm{I}}}}}}}_{490}}$$

DPH anisotropy measurements were taken by excitation at 358 nm and emission was collected at 430 nm. Anisotropy was calculated using the following equation:

$$r=\frac{\,{{{{\rm{I}}}}}_{{{{{\rm{VV}}}}}}-{{{{{\rm{GI}}}}}}_{{{{{\rm{VH}}}}}}}{{{{{\rm{I}}}}}_{{{{{\rm{VV}}}}}}-{2{{{{\rm{GI}}}}}}_{{{{{\rm{VH}}}}}}}$$

I_VV_ and I_VH_ are the parallel and perpendicular polarised fluorescence intensities measured with vertically polarised excitation light. I_HV_ and I_HH_ are the same fluorescence intensities measured horizontally polarised light. G is the monochromator grating correction factor given by G = I_HV_/I_HH_. OriginPro version 8 was used to calculate statistical significance using a one-way ANOVA and the Tukey-Kramer post-hoc test with a value of *p* ≤ 0.05 to establish statistical significance.

### Microscopy and analysis

A fixative solution was prepared comprising 2.5% glutaraldehyde, 0.1 M PIPES buffer and 13.87 mM glucose dissolved in double glass distilled water. A wash buffer was prepared from the same reagents, excluding the glutaraldehyde. Four strains were selected for transmission electron microscopy analysis: WT, SmvR Δ106–109, PssA-D240G and the double mutant SmvR Δ106–109 PssA-D240G. Appropriate sub-inhibitory concentrations were determined to be 0.25× MIC for the single mutants and 0.125× MIC for the WT and double mutant.

Control and challenge samples were grown in 10 mL TSB or TSB with sub-inhibitory octenidine and pelleted at 4 °C. Pellets were then briefly washed with fixative before being fixed in fresh fixative solution overnight at 4 °C. Samples were osmicated in 1% osmium tetroxide in 0.1 M PIPES buffercontaining 13.87 mM glucose for 1.5 hours at 4 °C and then washed twice with distilled water. Samples were stained with 1% uranyl acetate in water for 1 h at room temperature and washed again in distilled water before dehydrating in a series of ethanol. Pre-infiltration with propylene oxide was carried out twice, both for 10 min before samples were infiltrated in a graded series of SPURR resin:propylene oxide mixture followed by infiltration of 100% SPURR resin for 24 h. Samples were then embedded and polymerised for 24 h at 60 °C. Images were acquired using a JEOL1400 plus microscope at 120 kV with a JEOL Ruby camera (JEOL, Japan).

The resulting images were analysed qualitatively as well as with an in-house MATLAB script leveraging the Image Processing Toolbox. Images were processed to include only in-plane bacteria, and the major axis length (cell length) was recorded for each cell. Histograms of cell length with fitted distributions were then generated using the MATLAB Statistics Toolbox.

### Reporting summary

Further information on research design is available in the Nature Research Reporting Summary linked to this article.

## Supplementary information

Supplementary Information

Reporting Summary

## Data Availability

The datasets generated and analysed during the current study are available in the BioProject (SubmissionID: SUB6110485, BioProject ID: PRJNA558315) and Metabolights repository (www.ebi.ac.uk/metabolights/MTBLS1681). Source data are presented in the Supplementary materials. All other data are available from the corresponding authors on reasonable request.
