# Supplementary material for: Pseudomonas aeruginosa adapts to octenidine via a combination of efflux and membrane remodelling
Source: Commun Biol. 2021 Sep 9;4:1058. doi: 10.1038/s42003-021-02566-4 (PMC8429429; doi:10.1038/s42003-021-02566-4)
Supplement: Supplementary file 1 — Supplementary Information [file 42003_2021_2566_MOESM1_ESM.pdf]

Supplementary Information

Strain	SmvR (186aa)	PssA (271aa)	PgsA (186aa)	other	Fold increase in Oct MIC
NCTC 13437	151fs				16
372261	Δ106-109				8
CAS2	Δ97-85kb				16
CAS3	Δ106-109				32
CAS4	Δ30-39, Δ106-109			Putative tpnA repressor protein fs	16
GH12	Δ102, Δ106-109			LasR R180G (transcriptional regulator)	32
PAO1	75fs				16

Supplementary Table 1 Mutations present in *P. aeruginosa* strains passaged in increasing concentrations of octenidine followed by 10 passages on TSA in the absence of octenidine, analysed using whole genome sequencing of mixed populations surviving in the highest concentration of octenidine. Recurring mutations appear in block colour. fs frame shift after listed nucleotide

Strain	SmvR	PssA	PgsA	Fold increase in Oct MIC
NCTC 13437	L28Q (100)			64
372261	Δ79-103 (29), Δ102 (100)			64
CAS2	Δ106-109 (100)			64
CAS3	2x 106-109 (98)		G125S (9)	32
CAS4	Δ106-109 (100)	W177L (7), A233P (24), V237A (10), D240V (11), P241S (40)		16
GH12	64fs (76), 65fs (17), Δ106-109 (6)			64
PAO1	Δ106-109 (100)	V222G (100)		64

Supplementary Table 2 Repeat experiment showing mutations present in *P. aeruginosa* strains passaged in increasing concentrations of octenidine, analysed using population mode of BreSeq (P≤0.01) to establish % of population carrying mutation (in brackets). Recurring mutations appear in block colour. fs frame shift after listed nucleotide

Strain	SmvR	PssA	PgsA	Fold increase in Oct MIC
NCTC 13437	Δ7-13 (30), Δ106-109 (77)			64
372261	Δ106-109 (30)			64
CAS2	Δ106-109 (53), Δ103 (49), T112M (10), P113L (11), E114G (10), H115R (9)			64
CAS3	Δ106-109 (16)			32
CAS4	Δ106-109 (97), G43C (4)			32
GH12	64fs (68), 65fs (31)	V222G (100)		64
PAO1	Δ106-109 (100)	V222G (100)		64

Supplementary Table 3 Third repeat of experiments showing mutations present in *P. aeruginosa* strains passaged in increasing concentrations of octenidine, analysed using population mode of BreSeq (P≤0.01) to establish % of population carrying mutation (in brackets). Recurring mutations appear in block colour. fs frame shift after listed nucleotide

NCTC 13437		1	2	3	4	5	6	final
SmvR	K51R*		24	35	16		100	100
SmvR	Δ54-57		3	7				
SmvR	Δ106-109		76	79	93	100		
PssA	247-248 dupl		43					
PssA	D240E				23		100	100

372261		1	2	3	4	5	6	final
SmvR	R3C			13	6			100
SmvR	Δ8-12			5				
SmvR	Δ80-103			32				
SmvR	Δ102			36				
SmvR	Δ106-109			38	93			
SmvR	E117*			2				
SmvR	E136*			8				
SmvR	Δ144-146			5				
SmvR	149fs			3				
SmvR	123*					100	100	100

CAS2		1	2	3	4	5	6	final
SmvR	Δ43-52		26	35	60	93	98	100
SmvR	W26R		3	3				
SmvR	70fs		4	5				
SmvR	Δ102		2	2				
SmvR	Δ106-109		53	54	37	11	3	
SmvR	115fs		7	8				
SmvR	Δ144-146		7	8				
SmvR	V147E		9	7				
PssA	W177L				11			
PssA	D240G				41	92	98	100
PgsA	T58M				14			

CAS3		1	2	3	4	5		final
SmvR	Δ106-109		80	24	5			
SmvR	A143P		13	78	97	100		100
SmvR	Δ144-146		6					
PssA	V222G			100	100	100		100
PgsA	W176*			14				

CAS4		1	2	3	4	5	6	final
SmvR	15fs		3					
SmvR	Δ82-109		80	88	96	100	100	100
SmvR	Δ106-109		69	59	6		45	
SmvR	146fs				8			
PgsA	T58M						97	100

GH12		1	2	3	4	5		final
SmvR	Δ42-51		2	3				
SmvR	88fs			2				
SmvR	Δ106-109		89	87	100	100		100
PssA	R221W					3		
PssA	V222G				100	100		100

PAO1		1	2	3	4	5	6	final
SmvR	A34V		3					
SmvR	E96*		8	3				

**SmvR**	**Δ102**	24	5				
**SmvR**	**Δ102-105**	72	56	100	100	100	100
**SmvR**	**144-146 dupl**	17	17				
**SmvR**	**V44fs**		2				
**SmvR**	**W166***		2				
**PssA**	**P223L**			97	86		

Supplementary Table 4 Mutations present in *P. aeruginosa* strains passaged in six doubling concentrations of octenidine (starting concentration 2μg/mL), analysed using population mode of BreSeq (P≤0.01). SmvR, PssA and PgsA were analysed and the listed mutations found in each strain. The % of the population containing the population is shown for each passage. fs frame shift after listed amino acid; dupl duplication of listed amino acids; * premature stop codon

Day	SmvR (186aa)	PssA (271aa)	PgsA (186aa)	Octenidine MIC fold-increase over time point 0
**33**	A21G (97), E128D (97), D164E (94), A180P (97)			4
**61**	A21G (95), 78fs (74), Δ102 (5), Δ106-109 (8), E128D (96), Q163* (2), D164E (91), A180P (95)			8
**75**	A34V (18), R139P (1), 180fs (1)			0
**96**	Δ102 (96), Δ106-109 (7), 180fs (1)			4
**110**	78fs (76), Δ106-109 (4)			8

Supplementary Table 5 % of *P. aeruginosa* population containing mutations in *smvR*, *pssA* and *pgsA* following dosing of sink waste trap containing a mixed species biofilm with octenidine containing body wash as established by population mode BreSeq (P≤0.01) to establish % of population carrying mutation (in brackets). All values are compared to time point 0. Octenidine dosing was paused between days 65 and 90 (framed). Recurring mutations appear in block colour.

Mutation /gene	Oligo name	Oligo	% Primer efficiency	Purpose/ published name	Reference
PssA V222G	LBOL363	G*T*CGGTAAAGACCACGGCGAAGGCCAGCACCACGATAAGGATCGCCACGA<br>ACGGCcCCCGGCCGCGCAGGTCGAGATCCTTGAAGCTG*T*G		recombineering	this study
PssA D240G	LBOL364	G*T*AGGCAAGGAACAGCAACAGGAGGATGCGCGGCGGGcCGGTAAAGACC<br>ACGGCGAAGGCCAGCACCACGATAAGGATCGCCACGAAC*G*G		recombineering	this study
PssA D240E	LBOL470	T*A*GGCAAGGAACAGCAACAGGAGGATGCGCGGCGGcTCGGTAAAGACCA<br>CGGCGAAGGCCAGCACCACGATAAGGATCGCCACGAACG*G*C		recombineering	this study
PgsA T58M	LBOL386	C*T*TCCACCAGCAAGACCAGCGCAACCGCCACCATCAGCTTGTCCGCGACCG<br>GGTCGAGGAAGGCGCCGAACGGCaTGCTCTGGCCGAG*G*C		recombineering	this study
SmvR Δ106-109	LBOL365	A*A*CCGGCTGCGATACCACTGGTTGCTGCCCTCCAGGTGCTCGGGCGTCTGG<br>ATCAGCGCCGCCGCCTTGCTGCTGGACAACTCGTCCGAACGCTGGGT*C*G		recombineering	this study
smvR	LBOL436	CCCAGCGTTCGGACGAG		Generate product for meltcurve analysis to test successful 12bp deletion	this study
	LBOL437	GTTGCTGCCCTCCAGGTG			
smvR	LBOL458	GAAATGGGCATCACCAAGGG	100	qPCR	this study
	LBOL459	TTCGAACAGGCTGTCGTAGG			this study
smvA	LBOL464	GCTCAACGTGGACGAACAGA	100	qPCR	this study
	LBOL465	CTCATCAGGCTGCCGAGC			this study
pssA	LBOL444	TCGTGGCGATCCTTATCGTG	100	qPCR	this study
	LBOL445	GTATTGCACAGGACCGGAGG			this study
pgsA	LBOL597	CAACCTGGGCAAGTGGAAGA	104	qPCR	this study
	LBOL598	CAGCACGTAGCCGAGAATCA			this study
fabD	LBOL534	CCTGGAAGACGCCGATGTAT	85	qPCR	this study
	LBOL535	GCGATCACTACCTGCCCC			this study
rpoD	LBOL009	CGTCCTCAGCGGCTATATCG	100 (assumed)	PA0576	Chang et al (2005) Environ Sci Technol
	LBOL010	TTCTTCTTCCTCGTCGTCCTTC		PA0576	
smvR	LBOL193	TCTGCCGGAACATGGGATAA		gene amplification for Sanger Squencing	this study
	LBOL194	CGGACTTCTACAAGGCCAAGA			this study
pssA	LBOL191	CTTGCCTGGCGATCTGGTAT		gene amplification for Sanger Squencing	this study
	LBOL192	GAGGGTTTCGGACGTGACTT			this study
pgsA	LBOL315	CTACCTGAACGATGCCTCCC		gene amplification for Sanger Squencing	this study
	LBOL316	TGAGCTATTCCCGCATCGTC			this study

<u>Supplementary Table 6</u> Oligos used in this study for recombineering and qPCR.
* phosphorothioation of the two first and last bases

	NAL	DOX	MEM	PIP	CAZ	CIP	CHL
PAO1	>512	64	1	4-8	0.5-1	0.5	512
PssA V222G	>512	64	1	4	0.5	0.5	512
PssA D240G	>512	64	1	4	0.5	0.5	512
PssA D240E	>512	64	1	4	0.5	0.5	512
PgsA T58M	>512	64	1	4	0.5	0.5	512
SmvR Δ106-109	>512	64	1	4	0.5	0.5	512
SmvR Δ106-109 PssA V222G	>512	64	0.5	4	0.5	0.5	512
SmvR Δ106-109 PssA D240G	>512	64	0.5	2-4	0.5	0.5	512
SmvR Δ106-109 PssA D240E	>512	64	1	4	0.5	0.5	512
SmvR Δ106-109 PgsA T58M	>512	64	1	4	0.5	0.5	512

Supplementary Table 7 Minimum inhibitory concentrations of nalidixic acid (NAL), doxycycline (DOX), meropenem (MEM), piperacillin (PIP), ceftazidime (CAZ), ciprofloxacin (CIP) or chloramphenicol (CHL) on PAO1 and isogenic mutants. Data from 3 independent, replicate MIC determinations.

Mutation	qPCR target	RQ	RQ Min	RQ Max	P-Value
PgsA T58M	pgsA	1.0	0.9	1.0	0.96
PssA V222G	pgsA	1.7	1.5	1.9	0.20
PssA D240G	pgsA	1.4	1.2	1.7	0.31
PssA D240E	pgsA	1.6	1.3	1.9	0.25
PgsA T58M SmvR Δ106-109	pgsA	1.5	1.3	1.7	0.29
PssA V222G SmvR Δ106-109	pgsA	1.3	0.7	2.5	0.42
PssA D240G SmvR Δ106-109	pgsA	2.0	0.7	5.5	0.13
PssA D240E SmvR Δ106-109	pgsA	1.3	0.9	1.8	0.46
SmvR Δ106-109	pgsA	1.0	0.6	1.5	0.92
WT	pgsA	1.0	0.3	3.2	1.00
PgsA T58M	pssA	0.7	0.6	0.7	0.47
PssA V222G	pssA	2.1	1.5	2.8	0.24
PssA D240G	pssA	0.6	0.4	1.0	0.38
PssA D240E	pssA	1.4	1.1	1.8	0.53
PgsA T58M SmvR Δ106-109	pssA	1.3	0.6	3.0	0.60
PssA V222G SmvR Δ106-109	pssA	1.4	0.4	4.6	0.58
PssA D240G SmvR Δ106-109	pssA	0.7	0.2	2.6	0.62
PssA D240E SmvR Δ106-109	pssA	1.1	0.5	2.6	0.83
SmvR Δ106-109	pssA	0.8	0.7	1.1	0.73
WT	pssA	1.0	0.1	7.0	1.00
PgsA T58M	smvA	1.0	0.9	1.0	0.88
PssA V222G	smvA	1.5	1.1	2.2	0.25
PssA D240G	smvA	1.3	0.7	2.3	0.45
PssA D240E	smvA	1.4	1.1	1.8	0.30
**PgsA T58M SmvR Δ106-109**	**smvA**	**71.2**	6.3	804.1	**0.01**
**PssA V222G SmvR Δ106-109**	**smvA**	**34.6**	6.0	198.2	**0.00**
**PssA D240G SmvR Δ106-109**	**smvA**	**77.1**	39.2	151.6	**0.00**
**PssA D240E SmvR Δ106-109**	**smvA**	**68.1**	16.6	279.9	**0.00**
**SmvR Δ106-109**	**smvA**	**21.5**	12.4	37.3	**0.00**
WT	smvA	1.0	0.3	3.1	1.00
PgsA T58M	smvR	0.6	0.5	0.9	0.53
PssA V222G	smvR	2.4	2.1	2.7	0.28
PssA D240G	smvR	2.2	2.0	2.5	0.32
PssA D240E	smvR	2.3	1.5	3.5	0.30
**PgsA T58M SmvR Δ106-109**	**smvR**	**20.5**	4.8	86.8	**0.02**
**PssA V222G SmvR Δ106-109**	**smvR**	**15.9**	2.7	92.4	**0.02**
**PssA D240G SmvR Δ106-109**	**smvR**	**26.0**	16.2	41.8	**0.03**
**PssA D240E SmvR Δ106-109**	**smvR**	**25.6**	8.9	73.4	**0.02**
**SmvR Δ106-109**	**smvR**	**9.7**	6.7	14.1	**0.06**
WT	smvR	1.0	0.1	12.8	1.00

Supplementary Table 8 ***smvA* and *smvR* are constitutively overexpressed in SmvR Δ106-109 mutants**. See methods section for details of qPCR method and analysis. Expression levels were compared to WT PAO1. RQ shows the fold change, RQ Min and Max show the error. Values with a significant P-value are highlighted in bold.

Mutation	qPCR target	RQ	RQ Min	RQ Max	P-Value
PgsA T58M	pgsA	0.9	0.8	1.1	0.51
PssA V222G	pgsA	1.2	0.9	1.6	0.19
PssA D240G	pgsA	1.3	1.1	1.4	0.13
PssA D240E	pgsA	1.1	0.8	1.6	0.41
**PgsA T58M SmvR Δ106-109**	**pgsA**	**1.4**	1.0	1.9	**0.07**
**PssA V222G SmvR Δ106-109**	**pgsA**	**1.8**	1.5	2.2	**0.01**
**PssA D240G SmvR Δ106-109**	**pgsA**	**2.6**	1.1	6.0	**0.02**
**PssA D240E SmvR Δ106-109**	**pgsA**	**6.9**	1.7	28.0	**0.02**
SmvR Δ106-109	pgsA	0.8	0.5	1.3	0.15
WT	pgsA	1.0	0.7	1.5	1.00
PgsA T58M	pssA	1.1	0.9	1.2	0.44
**PssA V222G**	**pssA**	**1.3**	1.2	1.5	**0.04**
**PssA D240G**	**pssA**	**0.5**	0.4	0.6	**0.00**
PssA D240E	pssA	1.0	0.8	1.3	0.85
**PgsA T58M SmvR Δ106-109**	**pssA**	**2.3**	1.0	5.2	**0.04**
**PssA V222G SmvR Δ106-109**	**pssA**	**2.6**	1.8	3.8	**0.00**
PssA D240G SmvR Δ106-109	pssA	0.7	0.4	1.4	0.16
**PssA D240E SmvR Δ106-109**	**pssA**	**7.5**	2.8	20.2	**0.01**
SmvR Δ106-109	pssA	0.8	0.6	1.2	0.20
WT	pssA	1.0	0.8	1.3	1.00
PgsA T58M	smvA	1.0	0.1	11.7	1.00
PssA V222G	smvA	0.7	0.4	1.3	0.68
PssA D240G	smvA	0.7	0.4	1.3	0.66
PssA D240E	smvA	0.6	0.4	1.0	0.52
**PgsA T58M SmvR Δ106-109**	**smvA**	**31.2**	14.3	68.5	**0.03**
**PssA V222G SmvR Δ106-109**	**smvA**	**35.4**	24.5	51.0	**0.03**
**PssA D240G SmvR Δ106-109**	**smvA**	**28.7**	16.9	48.9	**0.03**
**PssA D240E SmvR Δ106-109**	**smvA**	**126.4**	105.8	151.0	**0.02**
**SmvR Δ106-109**	**smvA**	**9.8**	7.3	13.1	**0.07**
WT	smvA	1.0	0.1	15.3	1.00
PgsA T58M	smvR	1.0	0.1	7.7	0.95
PssA V222G	smvR	1.0	0.5	1.7	0.93
PssA D240G	smvR	0.9	0.5	1.5	0.86
PssA D240E	smvR	0.7	0.5	1.0	0.50
**PgsA T58M SmvR Δ106-109**	**smvR**	**13.6**	9.1	20.3	**0.02**
**PssA V222G SmvR Δ106-109**	**smvR**	**20.0**	11.5	34.7	**0.01**
**PssA D240G SmvR Δ106-109**	**smvR**	**18.7**	16.0	21.8	**0.02**
**PssA D240E SmvR Δ106-109**	**smvR**	**89.0**	39.4	200.8	**0.00**
**SmvR Δ106-109**	**smvR**	**7.7**	5.9	10.2	**0.04**
WT	smvR	1.0	0.2	6.3	1.00

Supplementary Table 9 **When exposed to 0.25 x MIC octenidine double mutants increase expression of *pssA* and *pgsA***. See methods section for details of qPCR method and analysis. Expression levels were compared to WT PAO1 exposed to 0.25 x MIC octenidine. RQ shows the fold change, RQ Min and Max show the error. Values with a significant P-value are highlighted in bold.

A) WT unC vs WT C	*pgsA*	*pssA*	*smvR*	*smvA*
**13437**	6.6	2.6		
**372261**	3.2			
**CAS2**	2.7		3.1	
**CAS3**	3.1			
**CAS4**	2.5	2.3		
**GH12**		1.9		
**PAO1**	1.6	2.9		

B) oct unC vs oct C	*pgsA*	*pssA*	*smvR*	*smvA*
**13437**	4.1	2.5		
**372261L**	3.9			
**372261S**				
**CAS2**	3.7			
**CAS3**				
**CAS4**				
**GH12**		0.5		
**PAO1**				

C) WT unC vs oct unC	*pgsA*	*pssA*	*smvR*	*smvA*
**13437**			6.6	74.4
**372261L**		1.5	11.1	49.3
**372261S**		1.4	19.4	33.5
**CAS2**			43.5	65.7
**CAS3**			31.8	42.0
**CAS4**			13.7	51.3
**GH12**		5.3	45.4	46.9
**PAO1**		11.9	134.5	587.0

D) WT C vs oct C	*pgsA*	*pssA*	*smvR*	*smvA*
**13437**				105.4
**372261L**			11.8	90.9
**372261S**			11.2	30.8
**CAS2**	1.8	0.2	34.4	57.6
**CAS3**	0.5		20.4	66.6
**CAS4**			15.6	87.0
**GH12**			27.9	29.9
**PAO1**		3.1	55.0	54.4

E) WT unC vs WT unC	*pgsA*	*pssA*	*smvR*	*smvA*
**13437**	1.0	1.0	1.0	1.0
**372261**				
**CAS2**				
**CAS3**				
**CAS4**				
**GH12**				
**PAO1**				

F) WT C vs WT C	*pgsA*	*pssA*	*smvR*	*smvA*
**13437**	1.0	1.0	1.0	1.0
**372261**	0.4			
**CAS2**	0.3			2.2
**CAS3**	0.4			
**CAS4**				0.3
**GH12**				0.2
**PAO1**	0.3			

G) oct unC vs oct unC	*pgsA*	*pssA*	*smvR*	*smvA*
**13437**	1.0	1.0	1.0	1.0
**372261L**	0.7	2.6		
**372261S**		2.5		
**CAS2**		0.4		
**CAS3**		3.0		
**CAS4**				
**GH12**		3.6		
**PAO1**		11.9		

H) oct C vs oct C	*pgsA*	*pssA*	*smvR*	*smvA*
**13437**	1.0	1.0	1.0	1.0
**372261L**				
**372261S**				
**CAS2**		0.1		
**CAS3**	0.3			
**CAS4**				
**GH12**				
**PAO1**				

Supplementary Table 10 ***smvR*, *smvA*, *pssA* and *pgsA* are expressed to similar levels in all WT strains in the absence of 0.25x MIC and *smvA/R* are constitutively highly overexpressed in all adapted strains (oct) compared to parent strains (WT).** All other changes in gene expression are strain dependent. Each WT unchallenged (unC) was compared to the same WT strain challenged (C) with 0.25x MIC octenidine (A), and each adapted challenged to unchallenged (B). The adapted strains were compared to their unchallenged parental strain in the absence (C) and presence of 0.25x MIC octenidine. All WT (E and F) and adapted (G and H) strains were also compared to NCTC 13437 in the presence (F and H) and absence (E and G) of octenidine. Only significant values are shown. Significance of values is indicated by shading (black $P \leq 0.01$, dark grey $P \leq 0.05$, light grey $P \leq 0.1$). Results of three technical repeats of three biological repeats using *fabD* and *rpoD* as reference genes taking primer efficiency into account were calculated in ExpressionSuite v1.1 using $\Delta\Delta C_T$ method. See Supplementary Table 6 for primer sequences.

	Mutation	Phenotype	octenidine MIC	octenidine MBC	chlorhexidine MIC	chlorhexidine MBC
PAO1			2	2	8	8- 16
PW3320	ΔsmvR		2	4- 8	16	32- 64
PW3318	ΔsmvA		1	1- 2	8	8
PW8747	ΔoprJ		2	4- 8	8	8
PW1783	ΔoprM		2	4	4	4
PW5186	ΔoprN		2	2	8	16
PW8139	ΔopmD		2	2	16	16
K767 (PAO1)			2	16	4 - 8	16
K1491	ΔmexR	MexAB OprM +++	2	32	4 - 8	64
K1523	ΔmexB	MexAB OprM -	2	8	2 - 4	8 - 32
K1525	ΔmexXY	MexXY OprM -	2	16	2 - 8	16 - 64
K1536	ΔnfxB	MexCD OprJ +++	2	16	4	16
K2153	unknown	Potential MexXY OprM +++	4 - 8	8	8 - 16	32
K2376	ΔmexS derivative of K2153	MexEF OprN +++	4	8	8	32 - 64
K2415	ΔmexZ	MexXY-OprM ++	2	16	4	16 - 32
K2733	ΔmexB ΔmexXY ΔmexCD-oprJ ΔmexF	MexAB OprM - MexXY - MexCD OprJ -, MexEF-OprN-	2 - 32	2	8 - 64	16
K2946	ΔmexF derivative of K2153	MexEF OprN -	4	8	16 - 32	16 - 32
K2958	ΔmexCD	MexCD OprJ -	2 - 4	4	4	8

Supplementary Table 11 MIC and MBC of octenidine and chlorhexidine for transposon mutants of PAO1 in selected efflux pumps. Grey shading implies strains that are not PAO1 derivatives.

Strain	Sequence Type	Group†	Country of Isolation	Year	Specimen	Efflux pumps and regulators present in all strains, amino acid changes relative to PAO1						
						MexZ 210aa	MexR 147aa	NalC 213aa	NfxB 187aa	MexS 337aa	SmvA 501aa	SmvR 187aa
NCTC 13437	357	Group 2	U.K.	Pre 2008		L138R	V126E	S209R		D249N	A43T A98T V112L L278V F336L	
372261	244	Group 1	U.K.	2012	Water	H51R				A75V D249N	V112L V174A V175I T219A L278V F336L S350A	
CAS2	313	Group 1	U.K.	2015	Sputum		V126E	A145V S209R		D249N	A43T S95T A97T V112L V174A V175I T219A L278V F336L F414L	
CAS3	253	Group 2	U.K.	2015	BAL	L138R N186N	V126E	A145V S209R	R21H D56G	D249N	V112L L278V F336L	
CAS4	Novel	Group 1	U.K.	2015	Wound			S209R		D249N	V112L L278V F336L A446T	A180P
GH12	235	Group 2	U.K.	Not known	Sputum	V48A	K44M V126E	E153Q S209R		D249N	V112L L278V F336L	Q11H
PAO1	549	Group 1	Reference strain from Uni of Washington transposon library									

Supplementary Table 12. Strains used in this study and data related to mutations in various RND-family efflux pumps and their regulators. No changes were seen in MexT. †Group designations relate to the comparative genomic characterization as carried out by Freschi et al 2018[1], aa amino acids

HR-MAS NMR				
Model	Q^2		Permutated Q^2	
	Unchallenged	Octenidine exposed	Unchallenged	Octenidine exposed
Single recombinant mutants				
WT v PssA D240E	0.801	0.915	-0.171	-0.391
WT v PssA D240G	0.899	0.871	-0.554	-0.329
WT v PssA V222G	0.915	0.899	-0.312	-0.312
WT v PgsA T58M	0.921	0.888	-0.333	-0.128
WT v SmvR Δ106-109	0.849	0.866	-0.291	-0.234
Double recombinant mutants (SmvR Δ106-109 + X)				
WT v PssA D240E	0.911	0.915	-0.315	-0.455
WT v PssA D240G	0.899	0.901	-0.368	-0.341
WT v PssA V222G	0.851	0.899	-0.311	-0.376
WT v PgsA T58M	0.791	0.867	-0.401	-0.344
**Spent media**				
Single recombinant mutants				
WT v PssA D240E	0.511	0.522	-0.171	-0.391
WT v PssA D240G	0.661	0.511	-0.554	-0.329
WT v PssA V222G	0.044	0.786	-0.312	-0.312
WT v PgsA T58M	0.545	0.088	-0.333	-0.128
WT v SmvR Δ106-109	-0.015	0.015	-0.298	-0.264
Double recombinant mutants (SmvR Δ106-109 + X)				
WT v PssA D240E	0.911	0.965	0.886	0.911
WT v PssA D240G	0.899	0.915	0.864	0.900
WT v PssA V222G	0.867	0.921	0.871	0.914
WT v PgsA T58M	0.896	0.933	0.901	0.899
Wild type PAO1				
Fresh v WT	0.951	0.941	-0.445	-0.411
WT v WT	-	0.761	-0.251	-0.398

Supplementary Table 13 **Q^2 scores for cross-validated OPLS-DA models for NMR metabolomics analysis of recombinant adapted isolates.** Q^2 and permutated Q^2 values shown above for ^1H HR-MAS NMR and liquid state data comparison models. Octenidine exposure was at ¼ respective MIC. Single recombinant mutants contain mutant gene coding the specified mutant protein alone whilst double recombinant mutants contain mutant gene plus SmvR Δ106-109. Good models are produced in almost all cases, notably, however, the introduction of the SmvR Δ106-109 mutation alone produces only a very modest change in metabolism.

Model (parent vs octenidine adapted in each case)	Q^2		Permutated Q^2	
	Unchallenged	Octenidine exposed	Unchallenged	Octenidine exposed
**PAO1**	-0.459	0.561	-0.352	-0.311
**CAS2**	0.068	0.874	-0.253	-0.267
**CAS3**	0.631	0.743	-0.113	-0.288
**CAS4**	0.529	0.681	-0.264	-0.400
**372261 LCV**	0.945	0.675	-0.267	-0.431
**372261 SCV**	0.955	0.916	-0.333	-0.160
**GH12**	0.358	0.720	-0.421	-0.222
**NCTC 13437**	0.291	0.932	-0.444	-0.215

Supplementary Table 14 **Q^2 scores for cross-validated OPLS-DA models for NMR metabolomics analysis of octenidine adapted isolates.** Q^2 and permutated Q^2 values shown above for ^1H HR-MAS NMR data comparison models. Good models are produced in all cases when comparing HR-MAS spectra between the parent and adapted strains with the exception of PAO1 and CAS2 in the absence of octenidine; the constitutive change in metabolism associated with octenidine adaption is respectively undetectable or very modest for these two isolates.

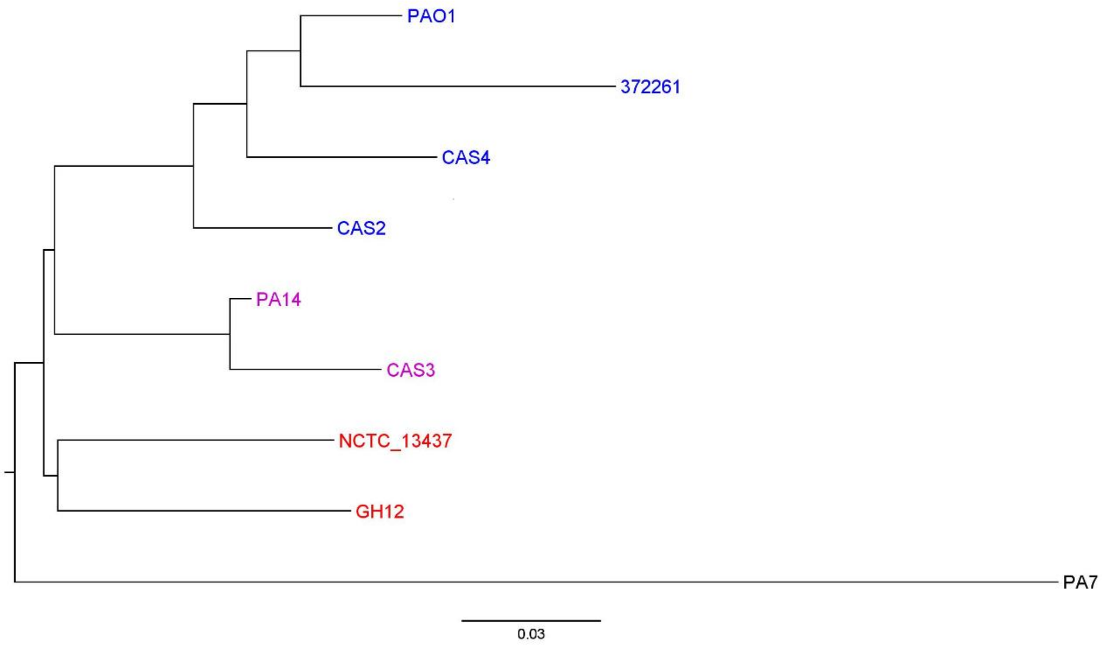

Supplementary Figure 1 **Whole genome phylogenetic analysis of the *Pseudomonas aeruginosa* strains used in this study.** Strains PA14 and PA7 are included as reference points. Strains have been colour coordinated dependent on potential grouping. The scale length indicates the number of nucleotide substitutions per nucleotide site.

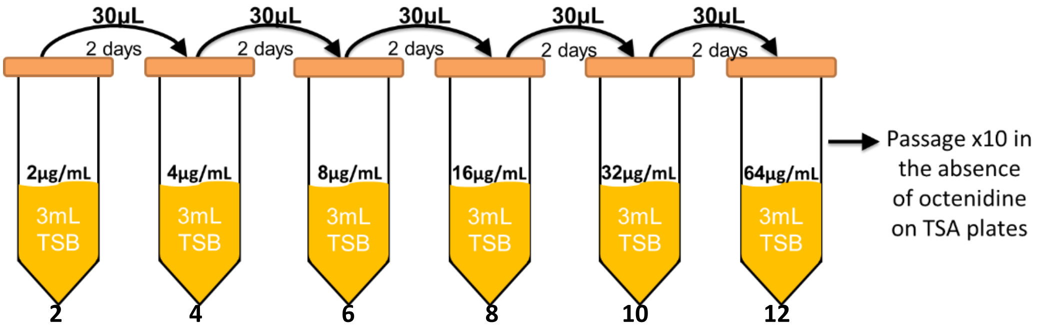

Day	2	4	6	8	10	12
**NCTC 13437**	8	16	16	64	64	128
**372261**	8	8	16	32	NG	NG
**CAS2**	8	16	16	32	64	64
**CAS3**	8	16	32	32	32	NG
**CAS4**	8	16	16	32	64	64
**GH12**	8	16	16	32	32	NG
**PAO1**	8	16	32	32	32	32

Supplementary Figure 2 **Schematic showing adaptation protocol used for selection of octenidine adapted strains**, as described previously in Shepherd *et al* 2018[2] and increases in MIC for populations as assessed during exposure. 7 parental strains were grown at 37˚C 250rpm in 3mL TSB containing 2ug/mL octenidine. Every two days the culture was refreshed by a 1:100 dilution into fresh media containing double the amount of octenidine for 12 days. Population samples were taking at all time points and analysed using BreSeq and population MICs. Single colonies were taken from these populations. This experiment was repeated to test whether adaptation was strain dependent. NG no growth

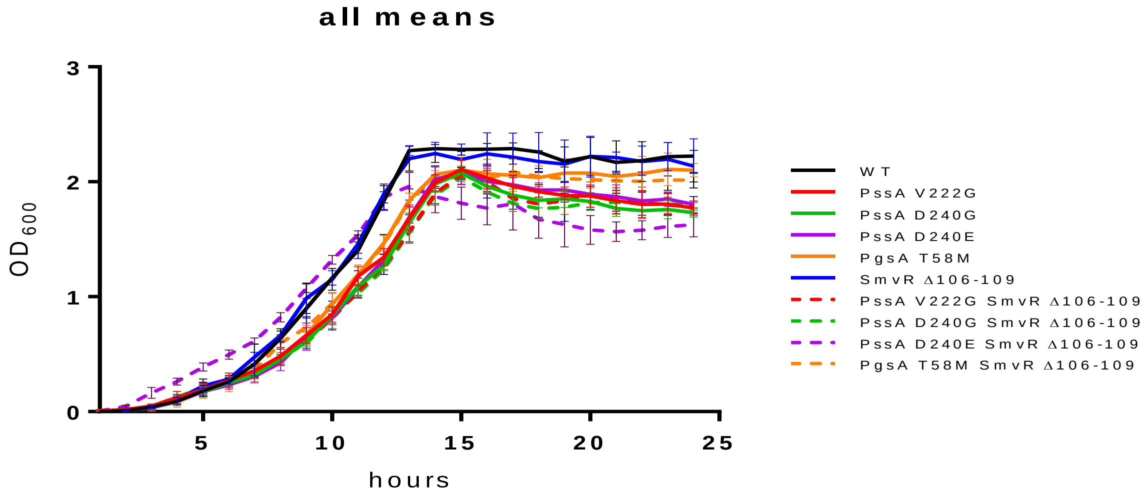

Supplementary Figure 3 **No significant difference in growth for PssA and PgsA mutants compared to WT and SmvRΔ106-109 single mutants**. *P. aeruginosa* PAO1 isogenic mutants were assessed for growth in TSA media over 24 hours at 37°C. The graph shows mean data of three technical repeats with standard error.

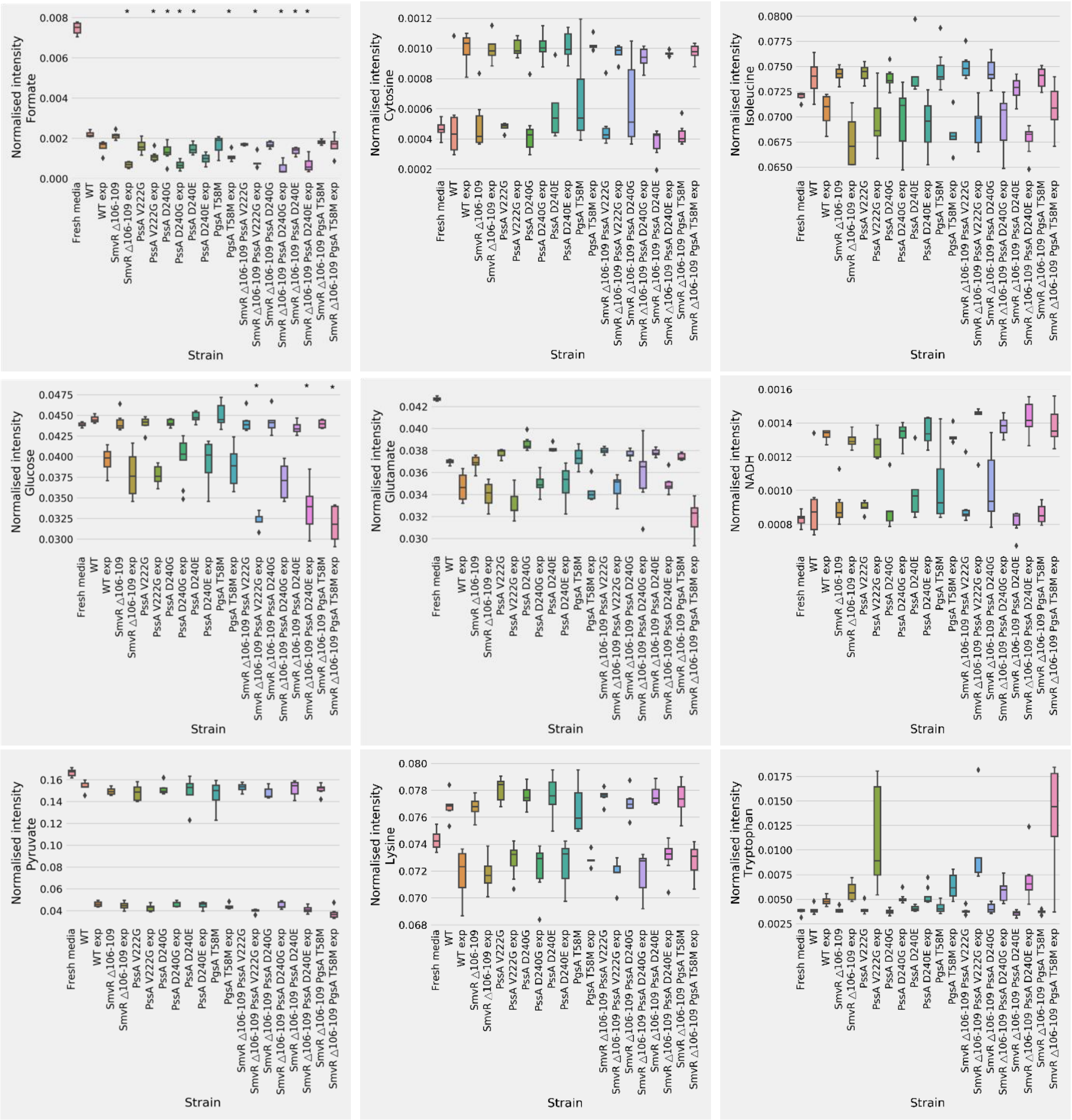

<u>Supplementary Figure 4</u> **Inducible response to octenidine challenge is independent of mutations in *P. aeruginosa* PAO1 and isogenic strains.** ^{1}H NMR was used to detect the concentrations of metabolites spent media after growth of recombinant octenidine resistant PAO1 strains in fresh tryptic soy broth (TSB) or TSB containing ¼ MIC of octenidine (denoted by exp). A minimum of 6 individual colonies were grown in 6 individual broths to the stationary phase (OD~1.2). PQN normalised metabolite associated resonance intensities, proportional to metabolite concentration, are compared above. *denotes statistical significance as calculated by one-way ANOVA and Tukey-Kramer post-hoc test between strains going in similar antimicrobial exposure conditions.

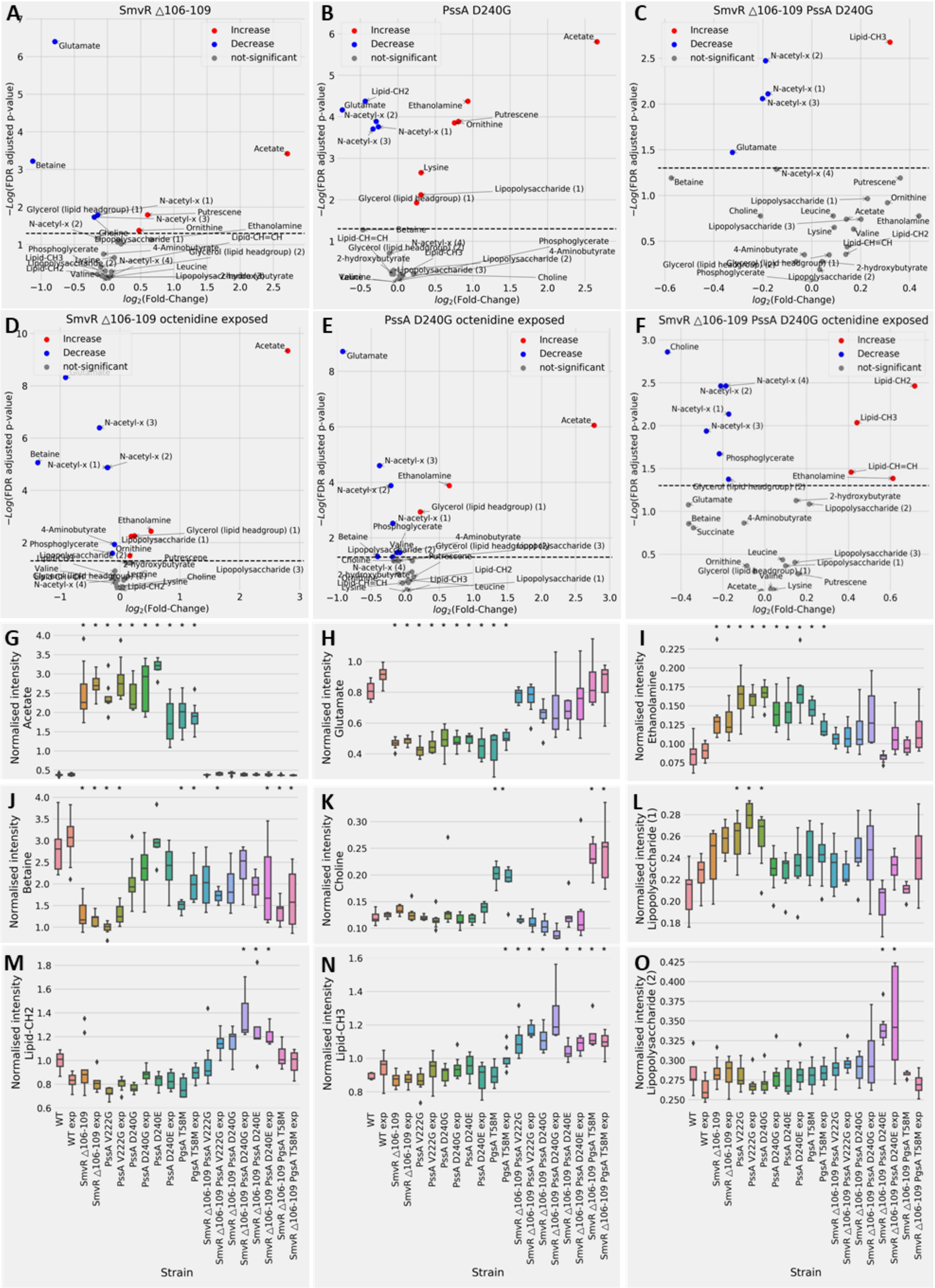

<u>Supplementary Figure 5</u> **Effect on *P. aeruginosa* PAO1 metabolism of isogenic mutations in the presence/absence of octenidine stress.** Volcano plots (A-F) are shown for individual comparisons of ^{1}H HR-MAS NMR data obtained for wild type (WT) *P. aeruginosa* PAO1 and each of the indicated conditions to reveal the effect of mutations in SmvR and/or PssA (D240G). Octenidine exposure was at ¼ respective MIC. Volcano plots are of PQN normalised data and allow comparison of fold changes and significance for each metabolite. Univariate analysis (G-O) of selected relative metabolite levels across all isogenic mutants shows significant differences with respect to WT, as determined by one-way ANOVA with Tukey's post hoc test (*).

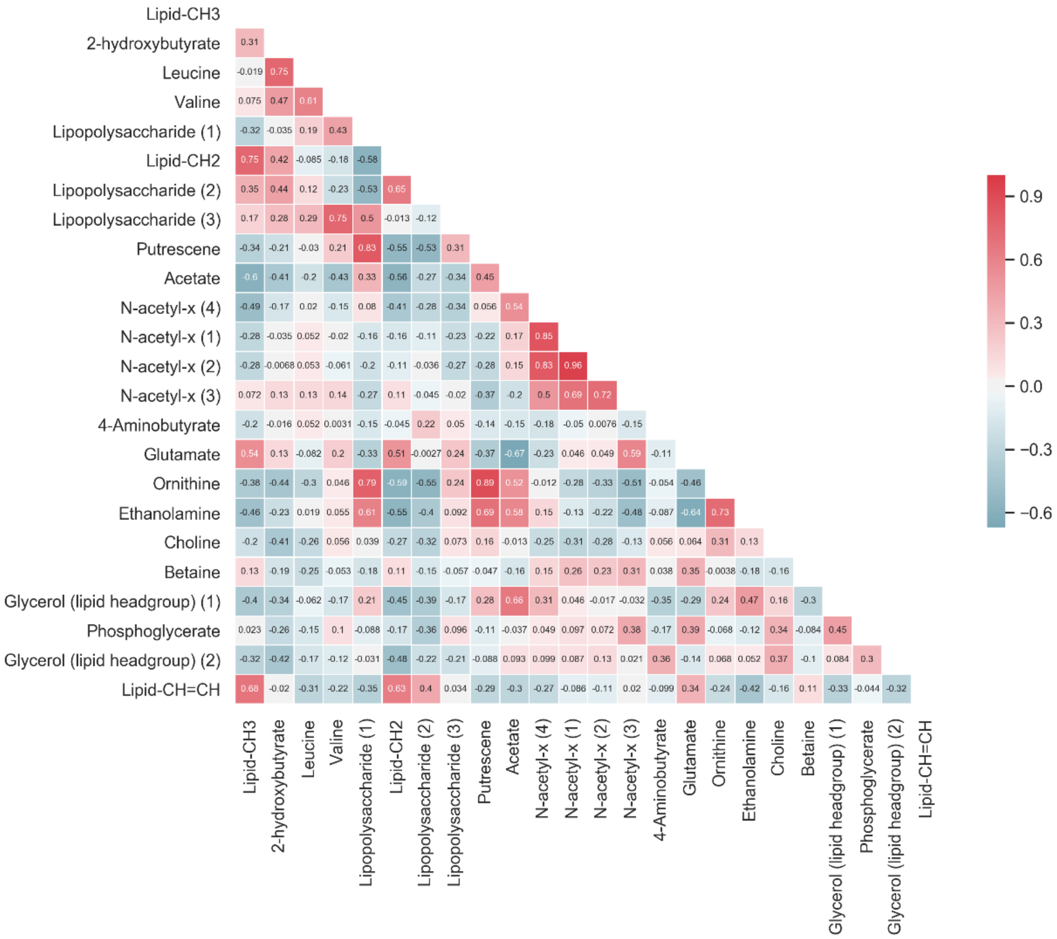

<u>Supplementary Figure 6</u> **Interrelationship matrix of assigned metabolites.** Spearman correlations between metabolite associated H[1] HR-MAS NMR resonances show relationships between intracellular metabolite concentrations. Correlations are representative of intracellular metabolite concentrations in all PA01 recombinant strains grown in both TSB and TSB containing ¼ MIC octenidine.

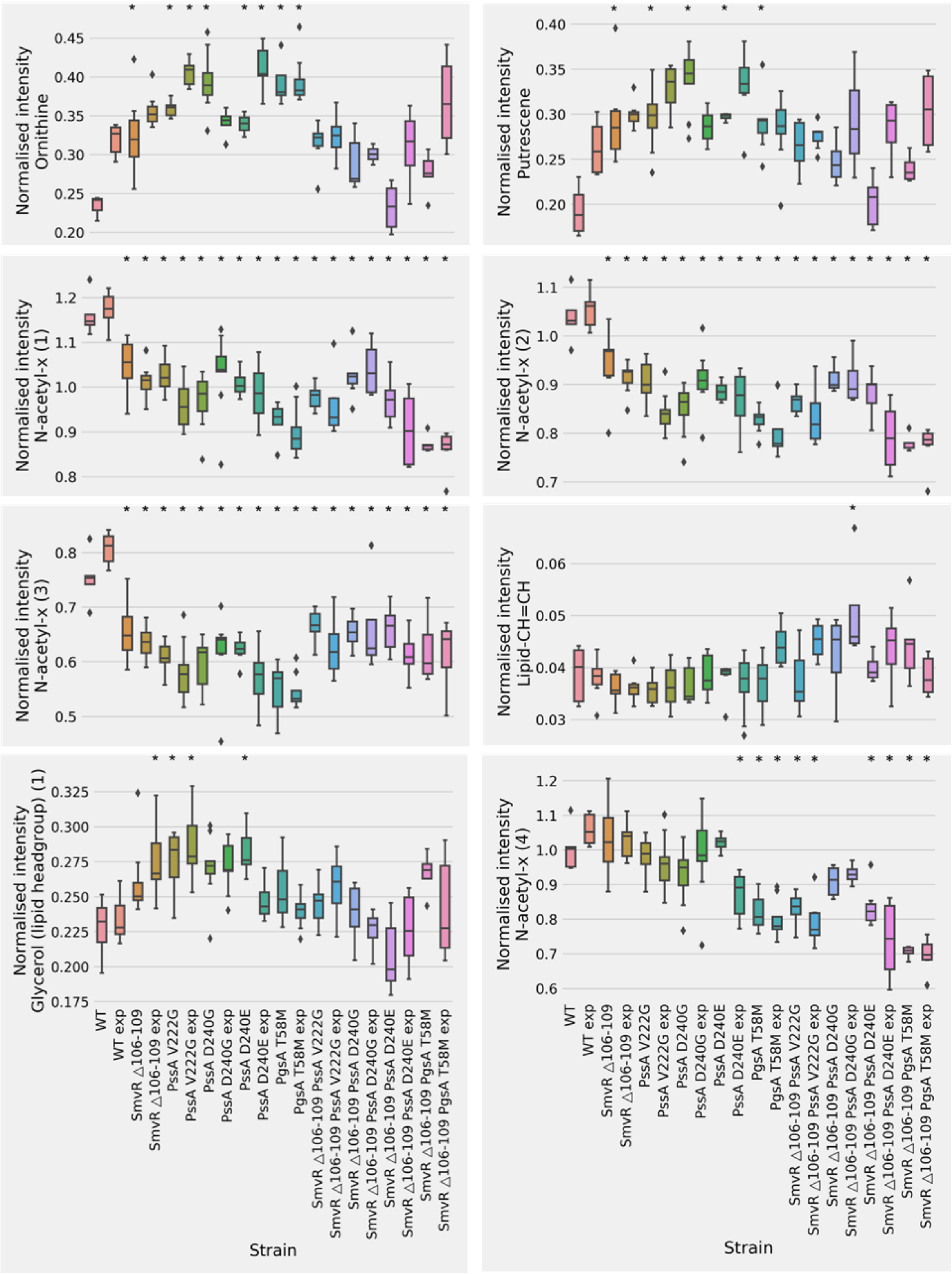

Supplementary Figure 7 **Univariate analysis of relative metabolite levels in *P. aeruginosa* PAO1 and isogenic strains in the presence (exp)/absence of octenidine stress.** Recombinant PA01 strains were grown in either TSB or TSB containing ¼ MIC (denoted by exp). For each strain a minimum of 6 individual colonies were grown in 6 separate broths to an optical density of ~1.2. Significant differences with respect to the results for the wild type (WT), or WT exp when strain is denoted with exp, was determined by one-way ANOVA with Tukey's post hoc test, are indicated (* p≤0.05). Other significant differences are described in the text. Octenidine exposure was at ¼ respective MIC.

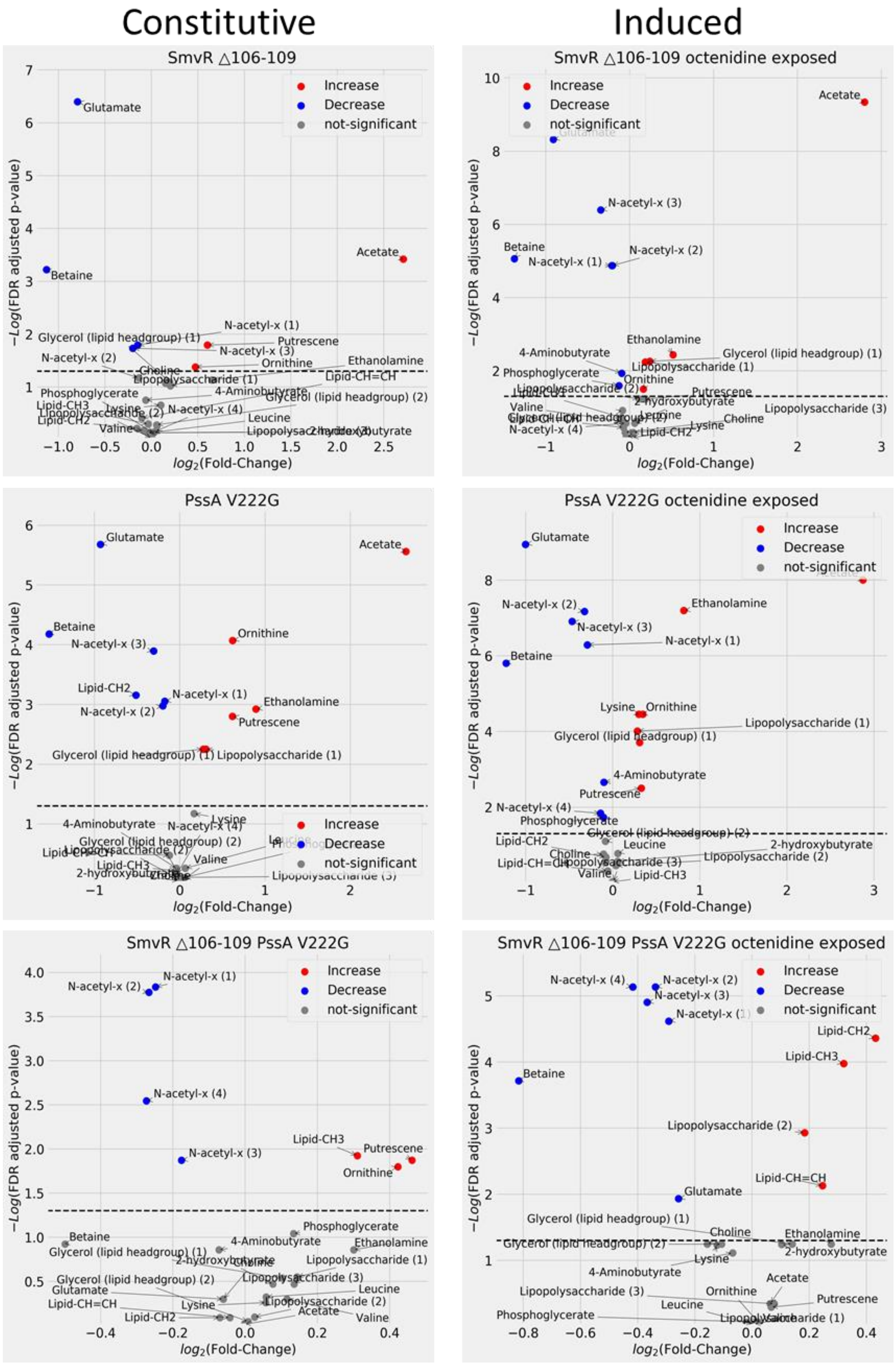

Supplementary Figure 8 **Effect on *P. aeruginosa* PAO1 metabolism of mutations in SmvR and/or PssA (V222G) in the presence/absence of octenidine stress.** Volcano plots are shown for individual comparisons of ^{1}H HR-MAS NMR data obtained for wild type (WT) *P. aeruginosa* PAO1 and each of the indicated conditions. Octenidine exposure was at ¼ respective MIC. Volcano plots are of PQN normalised data and allow comparison of fold changes and significance for each metabolite.

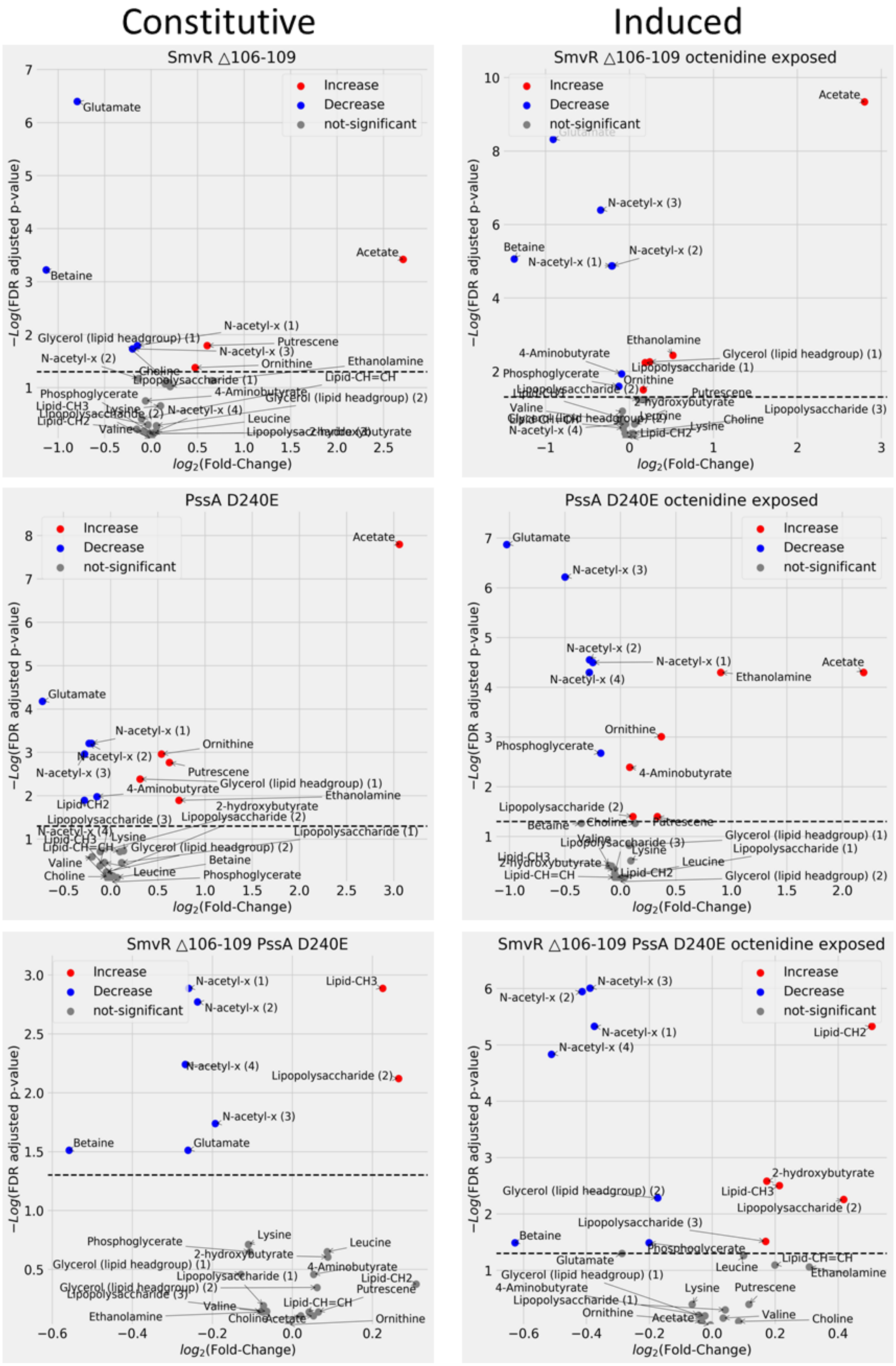

<u>Supplementary Figure 9</u> **Effect on *P. aeruginosa* PAO1 metabolism of mutations in SmvR and/or PssA (D240E) in the presence/absence of octenidine stress.** Fold change of H^1 HRMAS-NMR metabolite associated resonance intensity, proportional to metabolite concentration, between Wild-type and recombinant strain or octenidine exposed wild-type and recombinant strain is shown. For each strain a minimum of 6 biological replicates were carried out. Octenidine exposure was at ¼ respective MIC. Volcano plots are of PQN normalised data and allow comparison of fold changes and significance for each metabolite. A false discovery rate of 5% was applied to calculated P values.

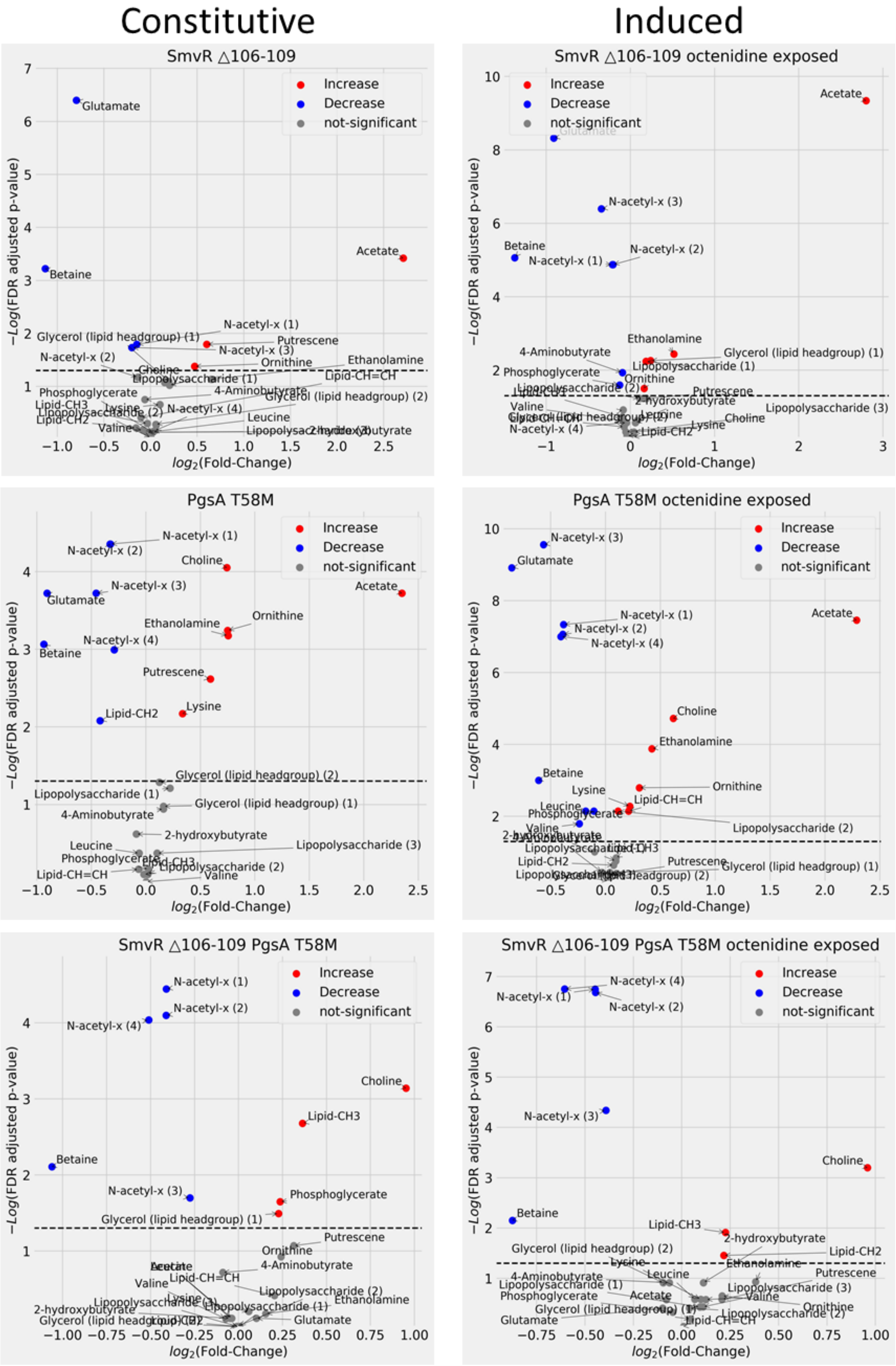

Supplementary Figure 10 **Effect on *P. aeruginosa* PAO1 metabolism of mutations in SmvR and/or PgsA (T58M) in the presence/absence of octenidine stress.** Fold change of H^1 HRMAS-NMR metabolite associated resonance intensity, proportional to metabolite concentration, between Wild-type and recombinant strain or octenidine exposed wild-type and recombinant strain is shown. For each strain a minimum of 6 biological replicates were carried out. Octenidine exposure was at ¼ respective MIC. Volcano plots are of PQN normalised data and allow comparison of fold changes and significance for each metabolite. A false discovery rate of 5% was applied to calculated P values.

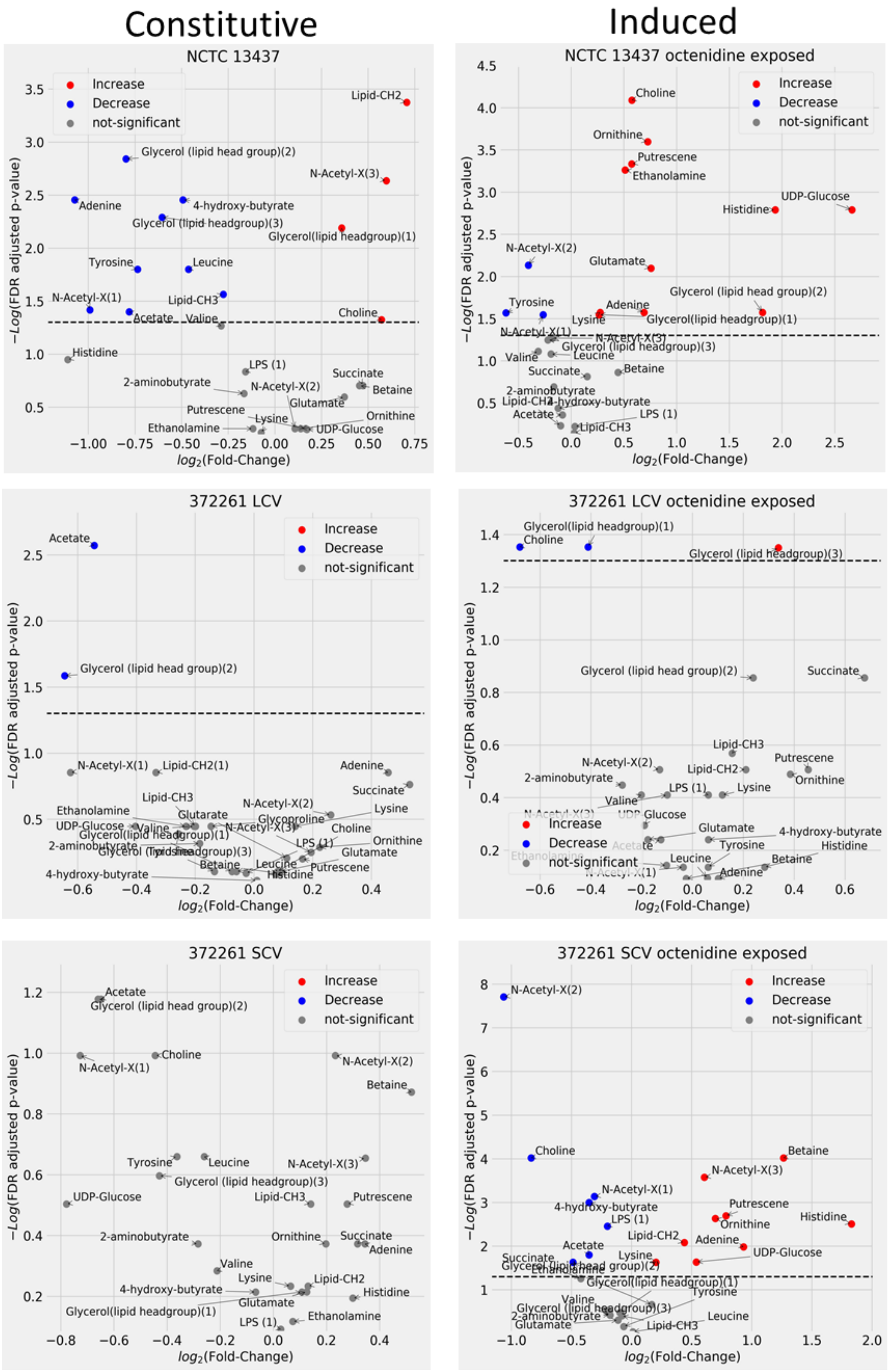

<u>Supplementary Figure 11</u> **Effect on cell metabolome of various *P. aeruginosa* isolates of adaptation to octenidine.** Fold change of H^1 HRMAS-NMR metabolite associated resonance intensity, proportional to metabolite concentration, between Wild-type and recombinant strain or octenidine exposed wild-type and recombinant strain is shown. For each strain a minimum of 6 biological replicates were carried out. Octenidine exposure was at ¼ respective MIC. Volcano plots are of PQN normalised data and allow comparison of fold changes and significance for each metabolite. A false discovery rate of 5% was applied to calculated P values.

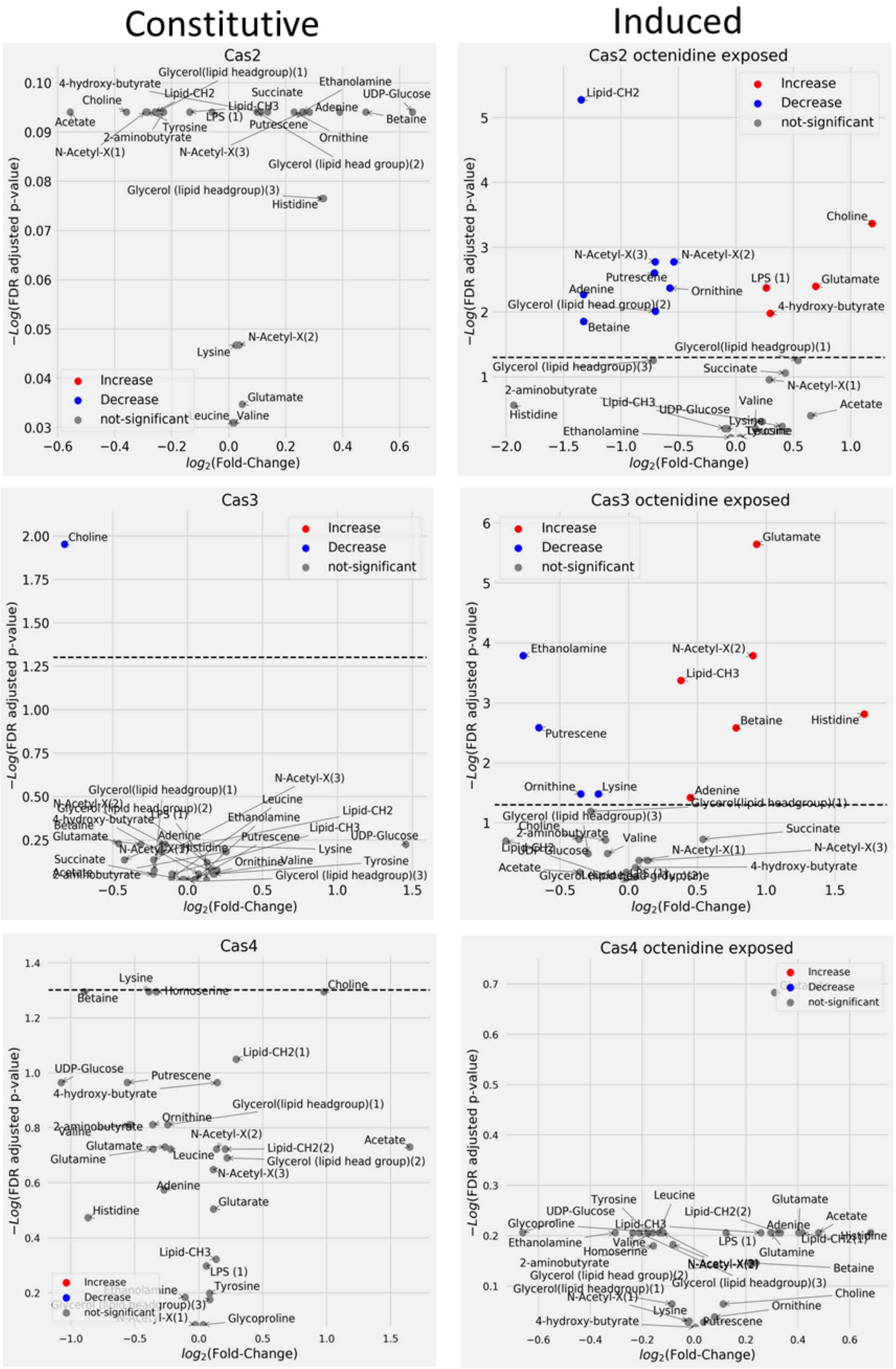

Supplementary Figure 12 **Effect on cell metabolome of various *P. aeruginosa* isolates of adaptation to octenidine.** Fold change of H^1 HRMAS-NMR metabolite associated resonance intensity, proportional to metabolite concentration, between Wild-type and recombinant strain or octenidine exposed wild-type and recombinant strain is shown. For each strain a minimum of 6 biological replicates were carried out. Octenidine exposure was at ¼ respective MIC. Volcano plots are of PQN normalised data and allow comparison of fold changes and significance for each metabolite. A false discovery rate of 5% was applied to calculated P values.

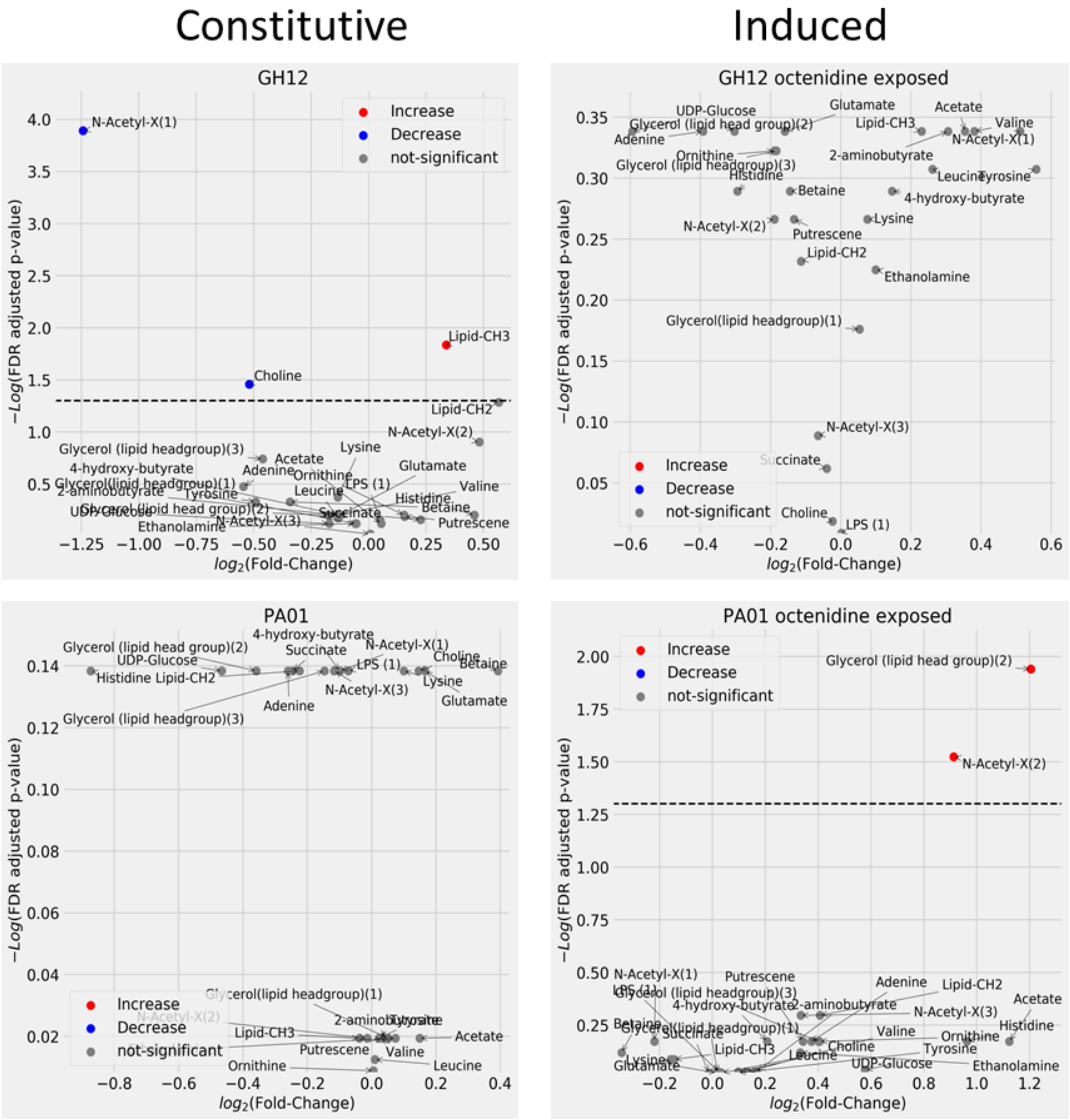

Supplementary Figure 13 **Effect on cell metabolome of various *P. aeruginosa* isolates of adaptation to octenidine.** Fold change of H^1 HRMAS-NMR metabolite associated resonance intensity, proportional to metabolite concentration, between Wild-type and recombinant strain or octenidine exposed wild-type and recombinant strain is shown. For each strain a minimum of 6 biological replicates were carried out. Octenidine exposure was at ¼ respective MIC. Volcano plots are of PQN normalised data and allow comparison of fold changes and significance for each metabolite. A false discovery rate of 5% was applied to calculated P values.

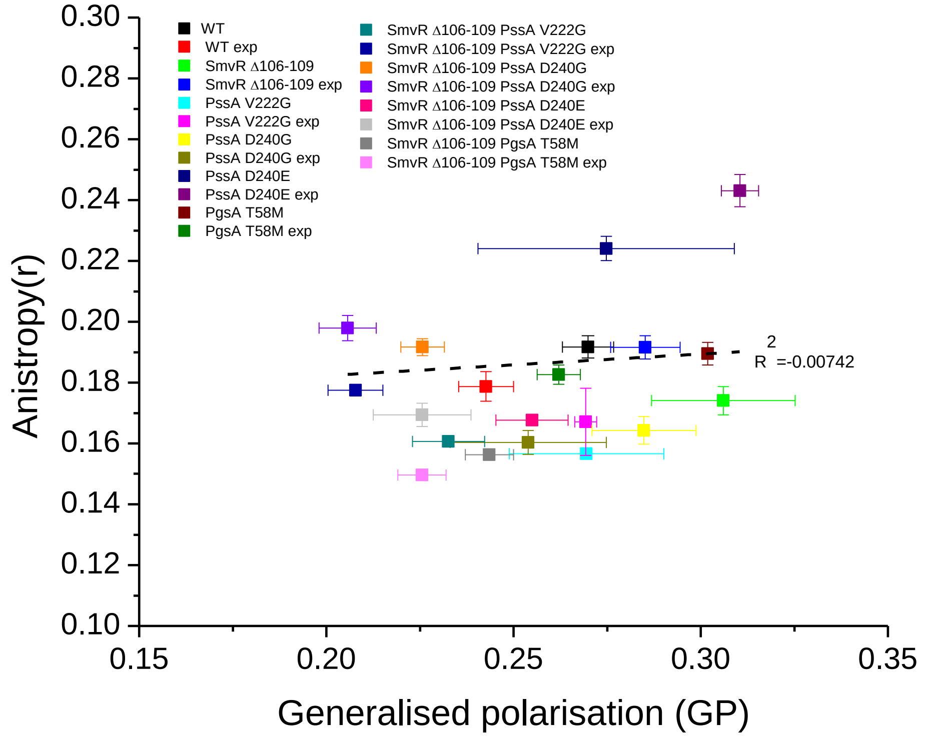

Supplementary Figure 14 **No correlation between average DPH fluorescence anisotropy and Laurdan GP measurements in *P. aeruginosa* PAO1 and isogenic strains in the presence (exp)/absence of octenidine stress.** Measures of lipid order – Laurdan GP, and lipid fluidity – DPH fluorescence anisotropy, in the absence or presence of octenidine (¼ MIC) when grown in tryptic soy broth are not correlated.

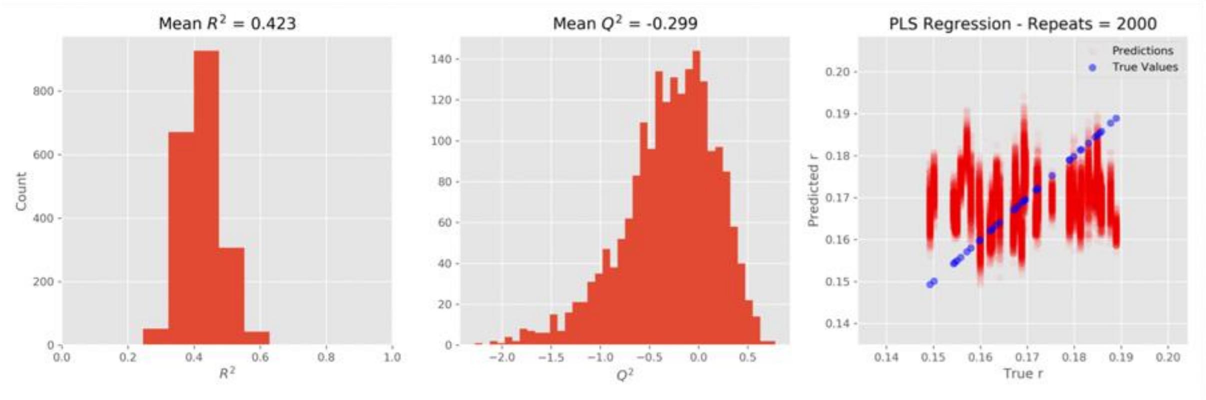

Supplementary Figure 15: Metabolic changes show poor correlation with lipid acyl chain order when detected by DPH fluorescence anisotropy. PLS regression model for correlation between DPH anisotropy fluorescence GP measurements and H_1 HR-MAS NMR metabolite resonance intensities of *P. aeruginosa* cell pellets are shown. This includes both wild type (PA01) and isogenic strains grown in either TSB or TSB containing ¼ MIC octenidine. Correlated DPH anisotropy and H_1 HRMAS measurements belong to the same cell pellet and each strain or condition had 9 biological replicates. For this model data was split into 70/30 training/test sets and used for Monte Carlo cross-validation models. The procedure was run 1000 times to avoid bias by sample separation and model performance was assessed through R^2 and Q^2 values. A negative Q^2 shows model cannot be used due to overfitting of data.

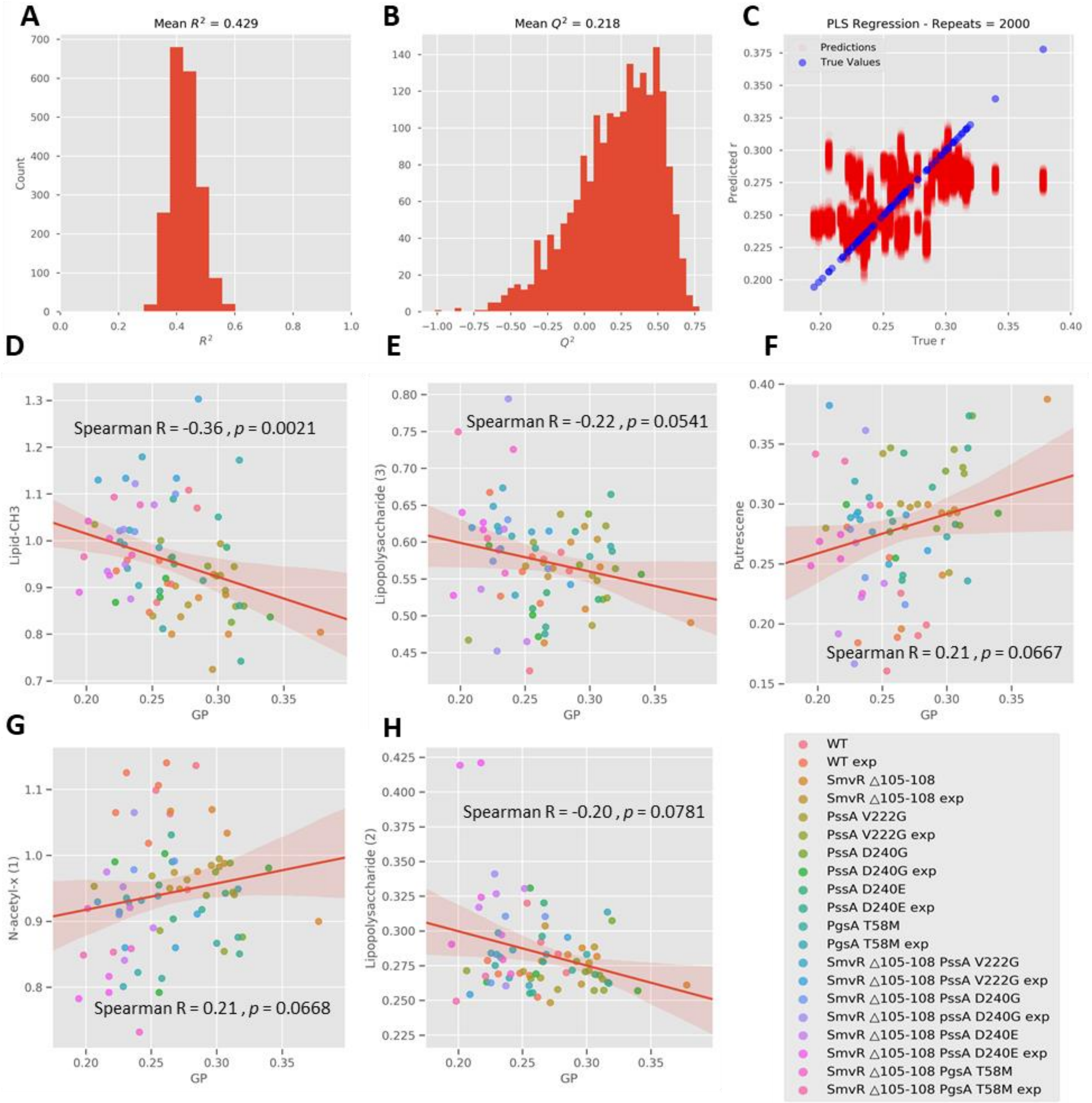

Supplementary Figure 16. PLS regression analysis of relative metabolite levels in *P. aeruginosa* PAO1 and isogenic strains in the presence (exp) /absence of octenidine stress. Measures of lipid order – Laurdan GP (**A**), and lipid fluidity – DPH fluorescence anisotropy (**B**) are shown for fluorescently labelled bacteria grown with or without 0.25x MIC octenidine in TSB. Spearman correlations from a PLS regression model are shown between Laurdan generalised-polarisation (GP) and ^{1}H HR-MAS NMR resonance intensity of intracellular metabolites or cell envelope components, which is proportional to their concentration. Recombinant PA01 strains were grown in TSB or TSB with ¼ MIC octenidine to the stationary phase (OD_{600} ~1.2). Each fluorescence and HR-MAS NMR measurement was carried out on the same biological replicate with a minimum of 6 biological replicates carried out for each condition. PLS regression models were validated by splitting data into 70/30 training/test sets for use with Monte Carlo cross-validation models. The procedure was run 1000 times to avoid bias by sample separation and model performance was assessed through R2 and Q2 values (Supplementary Table 12-S13).

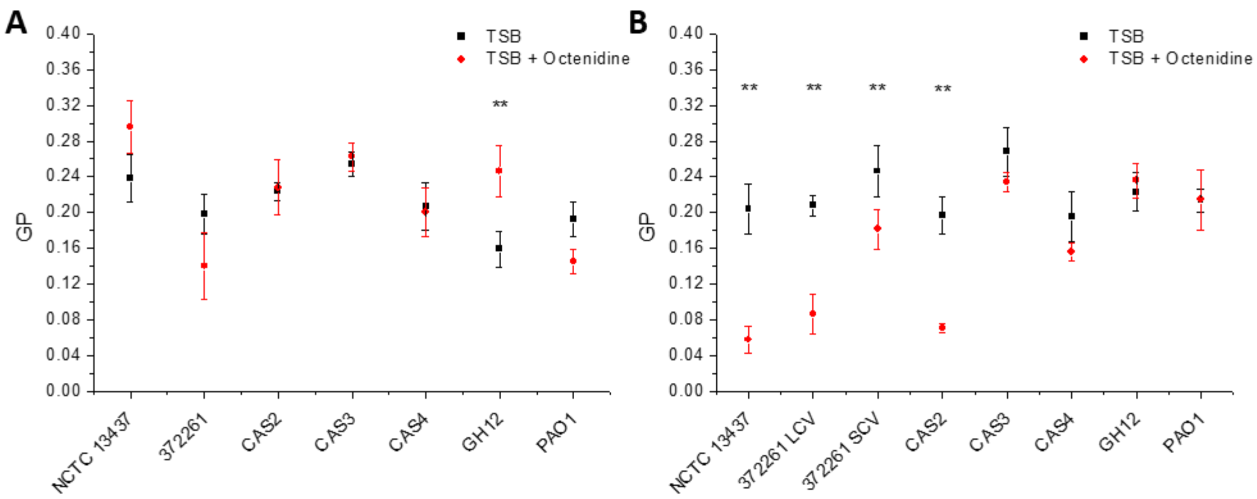

Supplementary Figure 17. Membrane disordering of octenidine adapted *P. aeruginosa* in the presence of octenidine stress. Measures of lipid order – Laurdan GP - in the absence or presence of octenidine (¼ MIC) when grown in tryptic soy broth are shown for parent (A) and octenidine adapted (B) *P. aeruginosa* isolates. Significance ($p < 0.05$) is shown between octenidine challenged and naïve conditions.

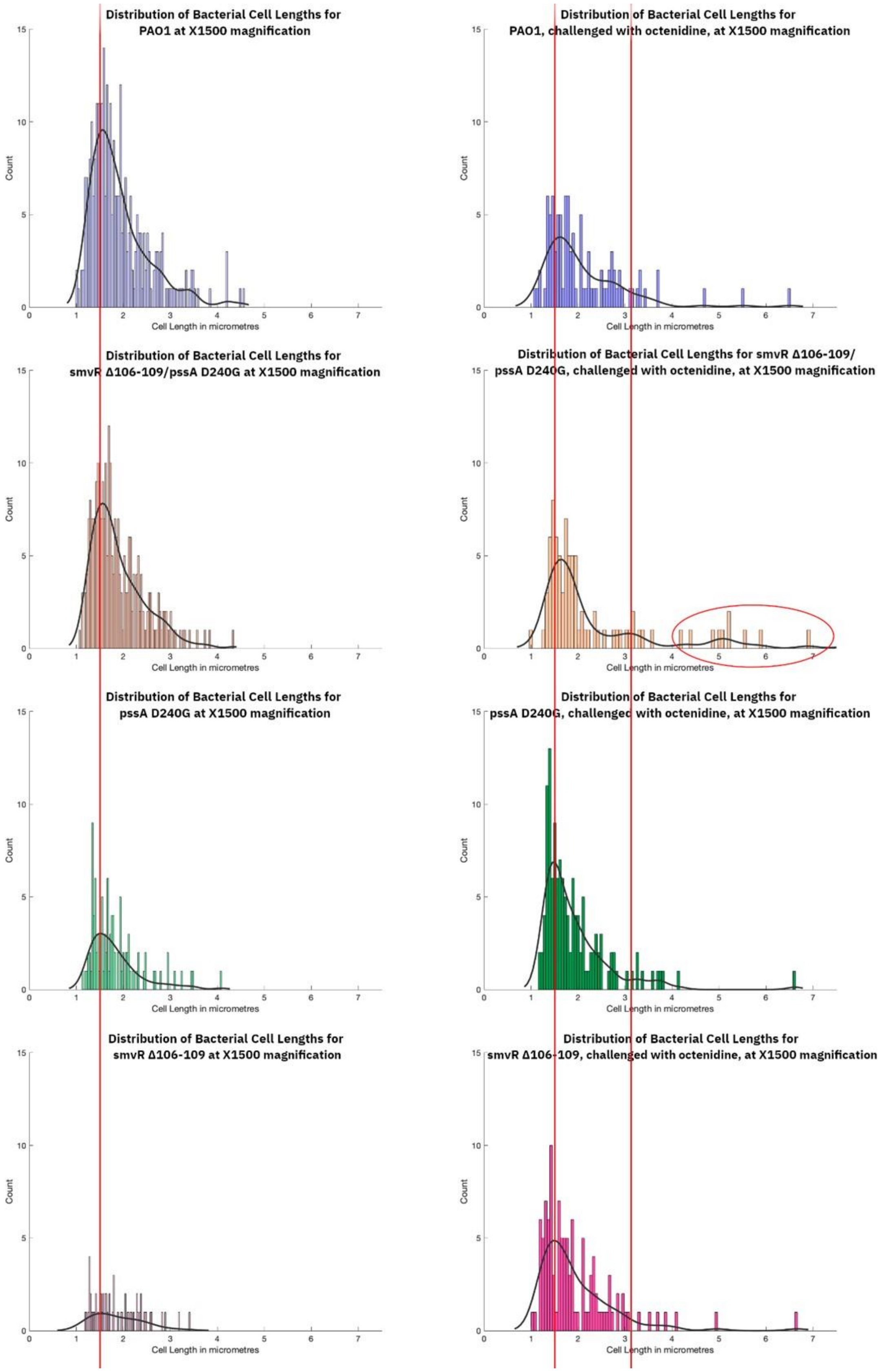

Supplementary Figure 18. Distribution of cell lengths from TEM of *P. aeruginosa* PAO1 and selected isogenic mutants in the presence or absence of octenidine stress. The distribution of cell lengths is shown for in-plane only bacteria observed in TEM obtained at x 1500 magnification.

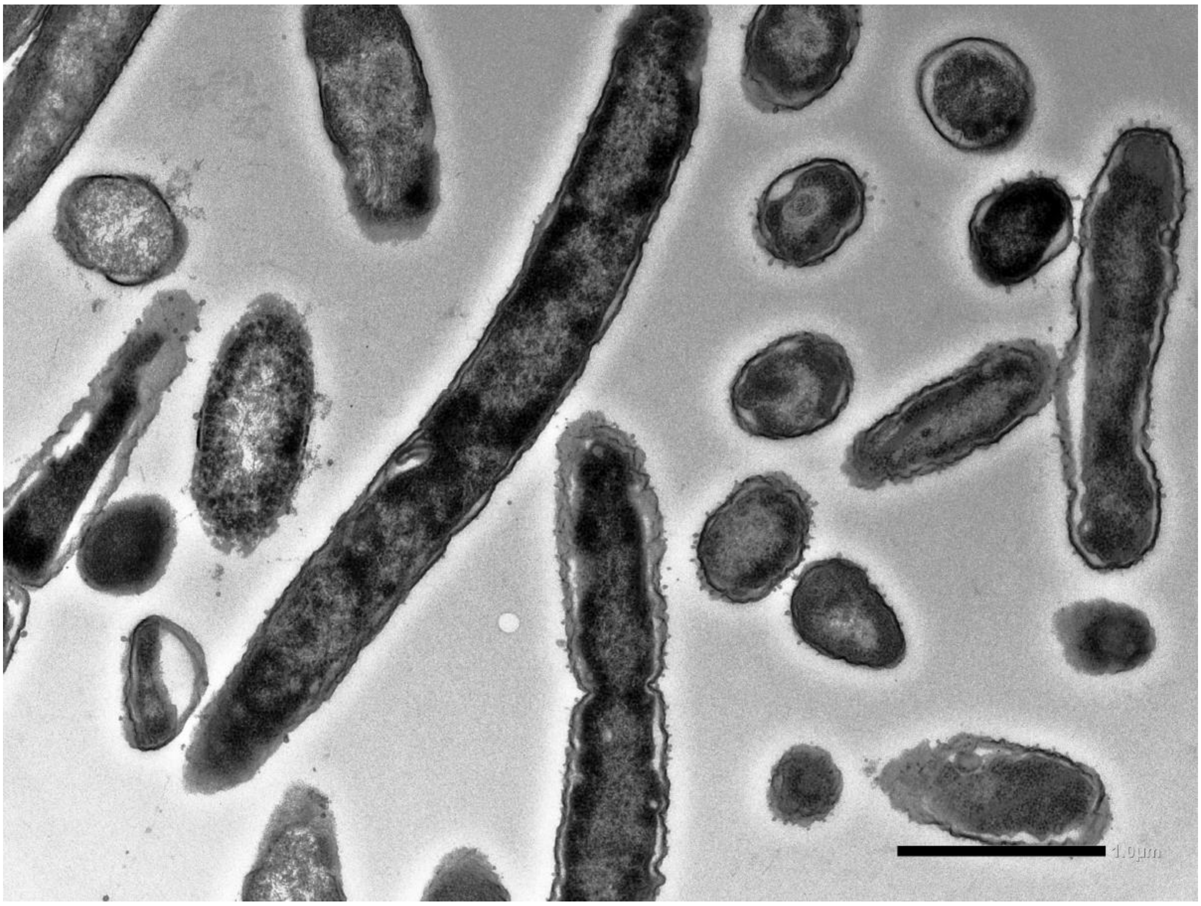

Supplementary Figure 19. Transmission Electron Micrograph of *P. aeruginosa* recombineered double mutant (PssA D240G & SmvR Δ106-109) cells in the presence of octenidine stress. This micrograph was acquired at x 10,000 magnification and shows an elongated cell as well as a cell with a septation defect.

1 Freschi, L. *et al.* Genomic characterisation of an international Pseudomonas aeruginosa reference panel indicates that the two major groups draw upon distinct mobile gene pools. *FEMS microbiology letters* **365**, doi:10.1093/femsle/fny120 (2018).
2 Shepherd, M. J., Moore, G., Wand, M. E., Sutton, J. M. & Bock, L. J. Pseudomonas aeruginosa adapts to octenidine in the laboratory and a simulated clinical setting, leading to increased tolerance to chlorhexidine and other biocides. *The Journal of hospital infection*, doi:10.1016/j.jhin.2018.03.037 (2018).
