# Supplementary material for: Pseudomonas aeruginosa adapts to octenidine via a combination of efflux and membrane remodelling
Source: Commun Biol. 2021 Sep 9;4:1058. doi: 10.1038/s42003-021-02566-4 (PMC8429429; doi:10.1038/s42003-021-02566-4)
Supplement: Supplementary file 2 — Reporting Summary [file 42003_2021_2566_MOESM2_ESM.pdf]

nature research

Corresponding author(s): J. Mark Sutton, Lucy J Bock

Last updated by author(s): 18/05/2021

Reporting Summary

Nature Research wishes to improve the reproducibility of the work that we publish. This form provides structure for consistency and transparency in reporting. For further information on Nature Research policies, see our Editorial Policies and the Editorial Policy Checklist.

Statistics

For all statistical analyses, confirm that the following items are present in the figure legend, table legend, main text, or Methods section.

n/a	Confirmed	
☐	☒	The exact sample size (*n*) for each experimental group/condition, given as a discrete number and unit of measurement
☐	☒	A statement on whether measurements were taken from distinct samples or whether the same sample was measured repeatedly
☐	☒	The statistical test(s) used AND whether they are one- or two-sided<br>*Only common tests should be described solely by name; describe more complex techniques in the Methods section.*
☐	☒	A description of all covariates tested
☐	☒	A description of any assumptions or corrections, such as tests of normality and adjustment for multiple comparisons
☐	☒	A full description of the statistical parameters including central tendency (e.g. means) or other basic estimates (e.g. regression coefficient) AND variation (e.g. standard deviation) or associated estimates of uncertainty (e.g. confidence intervals)
☒	☐	For null hypothesis testing, the test statistic (e.g. *F*, *t*, *r*) with confidence intervals, effect sizes, degrees of freedom and *P* value noted<br>*Give P values as exact values whenever suitable.*
☒	☐	For Bayesian analysis, information on the choice of priors and Markov chain Monte Carlo settings
☒	☐	For hierarchical and complex designs, identification of the appropriate level for tests and full reporting of outcomes
☒	☐	Estimates of effect sizes (e.g. Cohen's *d*, Pearson's *r*), indicating how they were calculated

Our web collection on statistics for biologists contains articles on many of the points above.

Software and code

Policy information about availability of computer code

Data collection	TopSpin (Bruker operating software) is used for NMR data acquisition. Sequence data was generated by Public Health England Genomic Services and Development Unit on an Illumina (HiSeq 2500). EM Images were acquired using a Jeol 1400 plus microscope at 120 kV with a Jeol Ruby camera (Jeol, USA). Cary Eclipse fluorescence data were obtained with the respective controlling software supplied with the instrument. Bacterial growth was measured using a Fluostar Omega Microplate Reader (BMG). Real time PCR data was acquired using a StepOne Plus from Applied Biosystems.

Data analysis	Origin 8.0 Pro was used for fluorescence analysis and Python modules (implementing published mathematical operations) for metabolomics data.<br>Genomic data was analysed from a minimum 150 Mb of Q30 quality data; FastQ files were quality trimmed using Trimmomatic. SPAdes 3.1.1 was used to produce draft chromosomal assemblies, and contigs of less than 1 kb were filtered out. Variant sequences were mapped to their parent strains using BWA 0.7.548. Bam format files were generated using Samtools and VCF files were constructed using GATK2 Unified Genotyper (version 0.0.7)50. They were further filtered using the following filtering criteria to identify high-confidence SNPs: mapping quality >30; genotype quality >40; variant ratio >0.9; read depth >10. All the above-described sequencing analyses were performed using PHE Galaxy Server. BAM files were visualized in Integrative Genomics Viewer (IGV) version 2.3.5552. BreSeq panalysis was carried out using protocol son the Barrick lab website (http://barricklab.org/twiki/pub/Lab/ToolsBacterialGenomeResequencing/documentation accessed between October 2017 and June 2019).<br><br>EM images were analysed using the Image Processing Toolbox.=, with data fitted using the MATLAB Statistics Toolbox. Differences in doubling time and intrinsic growth rate were calculated using growthcurver in R version 3.5.1.<br><br>ANOVA and other statistical analysis was performed in in GraphPad Prism version 6.04 for Windows, unless otherwise stated.<br><br>qPCR data was analysed using ExpressionSuite Software v1.1 from Applied Biosciences.

For manuscripts utilizing custom algorithms or software that are central to the research but not yet described in published literature, software must be made available to editors and reviewers. We strongly encourage code deposition in a community repository (e.g. GitHub). See the Nature Research guidelines for submitting code & software for further information.

Data

Policy information about availability of data

All manuscripts must include a data availability statement. This statement should provide the following information, where applicable:

- Accession codes, unique identifiers, or web links for publicly available datasets
- A list of figures that have associated raw data
- A description of any restrictions on data availability

The datasets generated and analysed during the current study are available in the BioProject (SubmissionID: SUB6110485, BioProject ID: PRJNA558315) and Metabolights repository (www.ebi.ac.uk/metabolights/MTBLS1681). The BioProject data is embargoed subject to publication.

Field-specific reporting

Please select the one below that is the best fit for your research. If you are not sure, read the appropriate sections before making your selection.

☒ Life sciences ☐ Behavioural & social sciences ☐ Ecological, evolutionary & environmental sciences

For a reference copy of the document with all sections, see nature.com/documents/nr-reporting-summary-flat.pdf

Life sciences study design

All studies must disclose on these points even when the disclosure is negative.

Sample size	Biological experiments (MICs, qPCR) were repeated a minimum of 3 times, as previous research has demonstrated this to be sufficient to detect accurate differences.
Data exclusions	No data was excluded
Replication	Replicate experiments were carried out where possible. Adaptation of bacterial populations in the drain rig was not replicated as this was a unique population which was disrupted as a consequence of the study. This data was used to confirm trends in replicate experiments of the lab adaptation studies.
Randomization	Randomisation was not an element of the study
Blinding	Analysis of EM samples was blinded prior to analysis to ensure unbiased assessment of the size of cells.

Reporting for specific materials, systems and methods

We require information from authors about some types of materials, experimental systems and methods used in many studies. Here, indicate whether each material, system or method listed is relevant to your study. If you are not sure if a list item applies to your research, read the appropriate section before selecting a response.

Materials & experimental systems

n/a	Involved in the study
x	Antibodies
x	Eukaryotic cell lines
x	Palaeontology and archaeology
x	Animals and other organisms
x	Human research participants
x	Clinical data
x	Dual use research of concern

Methods

n/a	Involved in the study
x	ChIP-seq
x	Flow cytometry
x	MRI-based neuroimaging
